# Dynamics of HIV-1/HTLV-I Co-Infection Model with Humoral Immunity and Cellular Infection

**DOI:** 10.3390/v14081719

**Published:** 2022-08-04

**Authors:** Noura H. AlShamrani, Matuka A. Alshaikh, Ahmed M. Elaiw, Khalid Hattaf

**Affiliations:** 1 Department of Mathematics, Faculty of Science, University of Jeddah, P.O. Box 80327, Jeddah 21589, Saudi Arabia; 2 Department of Mathematics, College of Science, Taif University, P.O. Box 11099, Taif 21974, Saudi Arabia; 3 Department of Mathematics, Faculty of Science, King Abdulaziz University, P.O. Box 80203, Jeddah 21589, Saudi Arabia; 4 Department of Mathematics, Faculty of Science, Al-Azhar University, Assiut Branch, Assiut 71524, Egypt; 5 Equipe de Recherche en Modélisation et Enseignement des Mathématiques (ERMEM), Centre Régional des Métiers de l’Education et de la Formation (CRMEF), Derb Ghalef, Casablanca 20340, Morocco

**Keywords:** HIV-1/HTLV-I co-infection, humoral immunity, cell-to-cell infection, global stability, Lyapunov function

## Abstract

Human immunodeficiency virus type 1 (HIV-1) and human T-lymphotropic virus type I (HTLV-I) are two retroviruses which infect the same target, CD$4^{+}$ T cells. This type of cell is considered the main component of the immune system. Since both viruses have the same means of transmission between individuals, HIV-1-infected patients are more exposed to the chance of co-infection with HTLV-I, and vice versa, compared to the general population. The mathematical modeling and analysis of within-host HIV-1/HTLV-I co-infection dynamics can be considered a robust tool to support biological and medical research. In this study, we have formulated and analyzed an HIV-1/HTLV-I co-infection model with humoral immunity, taking into account both latent HIV-1-infected cells and HTLV-I-infected cells. The model considers two modes of HIV-1 dissemination, virus-to-cell (V-T-C) and cell-to-cell (C-T-C). We prove the nonnegativity and boundedness of the solutions of the model. We find all steady states of the model and establish their existence conditions. We utilize Lyapunov functions and LaSalle’s invariance principle to investigate the global stability of all the steady states of the model. Numerical simulations were performed to illustrate the corresponding theoretical results. The effects of humoral immunity and C-T-C transmission on the HIV-1/HTLV-I co-infection dynamics are discussed. We have shown that humoral immunity does not play the role of clearing an HIV-1 infection but it can control HIV-1 infection. Furthermore, we note that the omission of C-T-C transmission from the HIV-1/HTLV-I co-infection model leads to an under-evaluation of the basic HIV-1 mono-infection reproductive ratio.

## 1. Introduction

Human immunodeficiency virus type 1 (HIV-1) is a retrovirus that attacks and infects healthy CD$4^{+}$ T cells; the crucial components of the human immune system. HIV-1 causes a fatal infectious disease called acquired immunodeficiency syndrome (AIDS). The World Health Organization (WHO) reported that there were about 36.7 million people living with HIV-1 at the end of 2016, and 1.8 million people become newly infected globally in 2016 [1]. In vivo, HIV-1 has two modes of dissemination, virus-to-cell (V-T-C) and cell-to-cell (C-T-C). In V-T-C dissemination, HIV-1 particles emitted from HIV-1-infected CD$4^{+}$ T cells search for new healthy CD$4^{+}$ T cells to infect. In the C-T-C mode of dissemination, HIV-1 can be transferred from HIV-1-infected CD$4^{+}$ T cells to healthy CD$4^{+}$ T cells via direct contact through the formation of virological synapses. Many studies have shown that HIV-1 propagation in the case of direct C-T-C dissemination is more efficient and potent than in the case of V-T-C transmission [2,3,4,5]. Sigal et al. [6] have reported that C-T-C dissemination of HIV-1 causes multiple infections of healthy CD$4^{+}$ T cells and then reduces the efficacy of drug therapies. Cytotoxic T lymphocytes (CTLs) and antibody immune responses are the two arms of the immune system. CTLs kill the HIV-1-infected CD$4^{+}$ T cells, whereas antibodies produced by B cells neutralize HIV-1 particles.

Human T-cell leukemia/lymphoma virus type I (HTLV-I) is a retrovirus that can cause the following diseases: HTLV-I-associated myelopathy (HAM), tropical spastic paraparesis (TSP) and adult T-cell leukemia/lymphoma (ATL). HTLV-I infects about 10–25 million people worldwide. Like HIV-1, HTLV-I infects healthy CD$4^{+}$ T cells. The infection is achieved through direct C-T-C contact between HTLV-I-infected CD$4^{+}$ T cells and healthy CD$4^{+}$ T cells [7]. The CTL immune response is an essential component for controlling HTLV-I infection through the lysis of HTLV-I infected CD$4^{+}$ T cells [8,9].

Both HIV-1 and HTLV-I can be transmitted from infected people to uninfected ones through sexual relationships, infected sharp objects, blood transfusions and organ transplantation. HTLV-I can also be transmitted via breastfeeding. HIV-1 and HTLV-I co-infected patients can be found in several geographical regions throughout the world, such as Europe, Japan, South America, the Caribbean, Mozambique and Brazil [10,11,12]. Isache et al. [13] have reported that the HTLV-I co-infection rate among HIV-1 infected people is 100 to 500 times higher in comparison with the general population. Co-infection with HTLV-I and HIV-1 may lead to faster progression to AIDS and the development of opportunistic infections [14].

Mathematical modeling and analysis of viral infection can be helpful in understanding the virus dynamics within a host, estimating different antiviral drug efficacies and predicting disease progression over the long term. Many researchers have made efforts to develop and analyze mathematical models of HIV-1 mono-infection, HTLV-I mono-infection and HIV-1/HTLV-I co-infection. We will outline some of these works in the remaining part of this section.

### 1.1. HIV-1 Mono-Infection Models

The primary and standard HIV-1 dynamics model developed in [15] comprises three compartments, healthy CD4${}^{+}$ T cells, active HIV-1-infected CD4${}^{+}$ T cells and free HIV-1 particles. In this model, it has been assumed that the HIV-1 infection is based only on V-T-C dissemination. This model has been modified to take into account the two modes of HIV-1 infection, V-T-C and C-T-C.

#### 1.1.1. HIV-1 Mono-Infection Models with C-T-C Dissemination

The HIV-1 dynamics model with both V-T-C and C-T-C dissemination can be formulated as:

(1) $$\left\{\begin{array}{c} \frac{dH}{dt}=\xi -\alpha H-\psi_{1}HP-\psi_{2}HI^{A},\\ \frac{dI^{A}}{dt}=\psi_{1}HP+\psi_{2}HI^{A}-\gamma I^{A},\\ \frac{dP}{dt}=\kappa I^{A}-\beta P, \end{array}\right.$$

where H=H(t), $I^{A}=I^{A}\left(t\right)$ and P=P(t) denote the concentrations of healthy CD$4^{+}$ T cells, active HIV-1-infected CD$4^{+}$ T cells and HIV-1 particles at time *t*, respectively. The HIV-1 virions are replicated via two mechanisms, V-T-C and C-T-C. The healthy CD$4^{+}$ T cells are created at a specific constant rate, ξ. The term $\psi_{1}HP$ denotes the V-T-C contact (incidence) rate between HIV-1 particles and healthy CD$4^{+}$ T cells. The term $\psi_{2}HI^{A}$ represents the C-T-C contact rate between active HIV-1-infected CD$4^{+}$ T cells and healthy CD$4^{+}$ T cells. $\kappa I^{A}$ represents the production rate of free HIV-1 particles from HIV-1-infected CD$4^{+}$ T cells. The terms αH, $\gamma I^{A}$ and βP are the death rates of compartments *H*, $I^{A}$ and *P*, respectively. This model was extended in [16] by assuming the logistic growth of healthy CD$4^{+}$ T cells. Mondal et al. [17] incorporated multi-drug therapies into the model (1) and studied the local and global stability of the steady states. Moreover, Pontryagin’s maximum principle has been used to determine optimal treatment regimens.

The modeling of HIV-1 infection with V-T-C and C-T-C modes of dissemination has attracted the attention of many researchers who have included additional biological mechanisms in model (1), such as:

- **Time delay models**: In reality, biological transitions such as infection interactions are not instantaneous but take time. In virology, intracellular delay accounts for the time of initial infection until the production of new virions. Lai and Zou [18] studied an HIV-1 infection model with C-T-C dissemination and two types of distributed time delays. Adak and Bairagi [19] investigated an HIV-1 infection model with C-T-C dissemination and discrete delays. They assumed logistic growth for healthy CD$4^{+}$ T cells and a saturated incidence rate for V-T-C infection in the form $\frac{\psi_{1}H^{n}P}{a^{n}\;+\;H^{n}}$, where n≥1 and a>0.
- **Latent infected cell models**: The impact of latent infected cells and antiretroviral therapy on the dynamics of HIV-1 with C-T-C dissemination was studied in [20]. Wang et al. [21] included latent infected cells and intracellular delays into their model of HIV-1 dynamics with C-T-C dissemination. In [20,21], both the local and global stability of steady states were investigated.
- **CTL immune response models**: Guo and Qiu [22] included the CTL immune response, latent infected cells and antiretroviral therapy in their model (1). Wang et al. [23] investigated the global stability of HIV-1 dynamics with C-T-C dissemination, a CTL immune response and a distributed delay. The model presented in [23] was generalized by Yan et al. [24], considering (i) two distributed delays and (ii) general functions for the V-T-C and C-T-C infection rates and the production/stimulation and removal of cells and HIV-1 particles. Elaiw and AlShamrani [25] investigated HIV-1 dynamics more generally in relation to the CTL immune response in cases where C-T-C dissemination is caused by both latent and active infected cells.
- **Diffusion models**: Ren et al. [26] addressed the effects of C-T-C dissemination and the mobility of viruses and cells on HIV-1 dynamics. Gao and Wang [27] investigated a reaction-diffusion HIV-1 dynamics model with delay and C-T-C dissemination. In [28], a diffusive viral infection model was developed, assuming that each latent and active infected cell collaborated in C-T-C infection. Sun and Wang [29] presented a diffusive HIV-1 infection model with C-T-C dissemination, assuming that the V-T-C infection rate could be expressed by a general function F(H,P).
- **Age-structured models**: Wang et al. [30] analyzed an age-structured HIV-1 infection model with C-T-C dissemination.

#### 1.1.2. HIV-1 Mono-Infection Model with C-T-C Dissemination and Humoral Immunity

The HIV-1 infection model with C-T-C dissemination and a humoral immune response can be formulated as:

(2) $$\left\{\begin{array}{c} \frac{dH}{dt}=\xi -\alpha H-\psi_{1}HP-\psi_{2}HI^{A},\\ \frac{dI^{A}}{dt}=\psi_{1}HP+\psi_{2}HI^{A}-\gamma I^{A},\\ \frac{dP}{dt}=\kappa I^{A}-\beta P-\pi BP,\\ \frac{dB}{dt}=\eta BP-\lambda B, \end{array}\right.$$

where B=B(t) denotes the concentration of HIV-1-specific antibodies at time *t*. The proliferation rate for HIV-1-specific antibodies is given by ηBP. The HIV-1 particles are neutralized by HIV-1-specific antibodies at a rate of πBP. The death rate of HIV-1-specific antibodies is represented by λB. Lin et al. [31,32] extended model (2) by considering an intracellular discrete-time delay. The effect of B-cell impairment on HIV-1 infection with C-T-C and a distributed time delay was investigated by Elaiw and Alshehaiween [33]. Guo et al. [34] extended model (2) by incorporating two intracellular discrete delays and both CTL and humoral immune responses.

### 1.2. HTLV-I Mono-Infection Models

The HTLV-I infection model, without including the effect of the immune response, can be expressed as [35]:

(3) $$\left\{\begin{array}{c} \frac{dH}{dt}=\xi -\alpha H-\psi_{3}HY^{A},\\ \frac{dY^{A}}{dt}=\tau \psi_{3}HY^{A}-\varphi Y^{A}, \end{array}\right.$$

where $Y^{A}=Y^{A}\left(t\right)$ is the concentration of active HTLV-I-infected CD4${}^{+}$ T cells. Healthy CD4${}^{+}$ T cells are infected with HTLV-I-infected CD$4^{+}$ T cells due to C-T-C dissemination at a rate of $\psi_{3}HY^{A}$. HTLV-I-infected CD$4^{+}$ T cells die at a rate of $\varphi Y^{A}$. $\tau \in \left(0,1\right)$ is the probability that new HTLV-I infections could enter a latent period. Several extensions of model (3) have been performed in many directions, including latent HTLV-I-infected CD$4^{+}$ T cells and leukemia (ATL) cells [36,37,38,39,40], the CTL immune response [41,42,43,44,45,46], time delays [47,48,49,50] and reaction-diffusion models [51].

### 1.3. HIV-1/HTLV-I Co-Infection Models

In recent works, Elaiw and AlShamrani [52,53,54] studied HIV-1/HTLV-I co-infection models with a CTL immune response. Alshaikh et al. [55] investigated HIV-1/HTLV-I co-infection models with humoral immunity by assuming that the healthy CD$4^{+}$ T cells were infected by HIV-1 only via V-T-C dissemination. Our aim in this paper was to develop an HIV-1/HTLV-I co-infection model with humoral immunity and both modes of HIV-1 infection, V-T-C and C-T-C taking into account both latent HIV-1-infected CD$4^{+}$ T cells and latent HTLV-I-infected CD$4^{+}$ T cells. We prove the nonnegativity and boundedness of the solutions of the models. We utilize the Lyapunov method to investigate the global stability of all steady states of the models. We illustrate the theoretical results with numerical simulations.

## 2. HIV-1/HTLV-I Co-Infection Model with Latent Infected Cells

In this section, we present the following system of ordinary differential equations (ODEs), which describe the interactions between seven compartments:

(4) $$\left\{\begin{array}{c} \frac{dH}{dt}=\xi -\alpha H-\psi_{1}HP-\psi_{2}HI^{A}-\psi_{3}HY^{A},\\ \frac{dI^{L}}{dt}=\left(1-\delta \right)\left(\psi_{1}HP+\psi_{2}HI^{A}\right)-\left(\varepsilon +\theta \right)I^{L},\\ \frac{dI^{A}}{dt}=\delta \left(\psi_{1}HP+\psi_{2}HI^{A}\right)+\varepsilon I^{L}-\gamma I^{A},\\ \frac{dY^{L}}{dt}=\tau \psi_{3}HY^{A}-\left(\rho +\varpi \right)Y^{L},\\ \frac{dY^{A}}{dt}=\rho Y^{L}-\varphi Y^{A},\\ \frac{dP}{dt}=\kappa I^{A}-\beta P-\pi BP,\\ \frac{dB}{dt}=\eta BP-\lambda B, \end{array}\right.$$

with initial conditions

(5) $$H\left(0\right)>0,\;I^{L}\left(0\right)\geq 0,\;I^{A}\left(0\right)\geq 0,\;Y^{L}\left(0\right)\geq 0,\;Y^{A}\left(0\right)\geq 0,\;P\left(0\right)\geq 0\;\mathrm{and}\;B\left(0\right)\geq 0,$$

where $I^{L}$ and $Y^{L}$ represent, respectively, latent HIV-1-infected CD$4^{+}$ T cells and latent HTLV-I-infected CD$4^{+}$ T cells. The terms $\varepsilon I^{L}$ and $\rho Y^{L}$ represent the activation rates of latent HIV-1-infected and latent HTLV-I-infected CD$4^{+}$ T cells, respectively. The fraction coefficient $\delta \in \left(0,1\right)$ is the probability that new HIV-1-infected CD$4^{+}$ T cells could be active and the remaining fraction 1−δ will be latent. The natural death rates of latent HIV-1-infected CD$4^{+}$ T cells and latent HTLV-I-infected CD$4^{+}$ T cells are demonstrated by $\theta I^{L}$ and $\varpi Y^{L}$, respectively. Table 1 summarizes the biological meanings of all variables and parameters.

Next, we will determine a bounded domain for the concentrations of the model’s compartments to ensure that our model is biologically acceptable. Particularly, the concentrations should not become negative or unbounded.

### 2.1. Properties of Solutions

**Lemma** **1.** *All Solutions $\left(H\left(t\right),I^{L}\left(t\right),I^{A}\left(t\right),Y^{L}\left(t\right),Y^{A}\left(t\right),P\left(t\right),B\left(t\right)\right)$ of system (4) with initial conditions (5) are nonnegative and ultimately bounded.*

**Proof.**  According to (4), we have

$$\begin{array}{cc} \frac{dH}{dt} & {|}_{H=0}=\xi >0,\;\;\;\;\frac{dI^{L}}{dt}{|}_{I^{L}=0}=\left(1-\delta \right)\left(\psi_{1}HP+\psi_{2}HI^{A}\right)\geq 0\;\mathrm{for}\;\mathrm{all}\;H,P,I^{A}\geq 0,\\ \frac{dI^{A}}{dt} & {|}_{I^{A}=0}=\delta \psi_{1}HP+\varepsilon I^{L}\geq 0\;\mathrm{for}\;\mathrm{all}\;H,P,I^{L}\geq 0,\;\;\;\;\frac{dY^{L}}{dt}{|}_{Y^{L}=0}=\tau \psi_{3}HY^{A}\geq \;\mathrm{for}\;\mathrm{all}\;H,Y^{A}\geq 0,\\ \frac{dY^{A}}{dt} & {|}_{Y^{A}=0}=\rho Y^{L}\geq 0\;\mathrm{for}\;\mathrm{all}\;Y^{L}\geq 0,\;\;\;\;\frac{dP}{dt}{|}_{P=0}=\kappa I^{A}\geq 0\;\mathrm{for}\;\mathrm{all}\;I^{A}\geq 0,\;\;\;\;\frac{dB}{dt}{|}_{B=0}=0. \end{array}$$

It follows from Proposition B.7 of [56] that $I^{L}\left(t\right),I^{A}\left(t\right),Y^{L}\left(t\right),Y^{A}\left(t\right),P\left(t\right),B\left(t\right)\geq 0$ for all t≥0 whenever the initial conditions (5) are satisfied.

Next, we aim to show the ultimate boundedness of the solutions. Form the first equation of system (4) we have $\frac{dH}{dt}\leq \xi -\alpha H$ and this implies that

$$\lim_{t\to \infty }\sup H\left(t\right)\leq \frac{\xi }{\alpha }=\Omega_{1}.$$

Let us define a function Ψ as:

$$\Psi =H+I^{L}+I^{A}+\frac{1}{\tau }\left(Y^{L}+Y^{A}\right)+\frac{\gamma }{2\kappa }P+\frac{\pi \gamma }{2\eta \kappa }B.$$

Then

$$\begin{array}{cc} \frac{d\Psi }{dt} & =\xi -\alpha H-\theta I^{L}-\frac{\gamma }{2}I^{A}-\frac{\varpi }{\tau }Y^{L}-\frac{\varphi }{\tau }Y^{A}-\frac{\gamma \beta }{2\kappa }P-\frac{\pi \gamma \lambda }{2\eta \kappa }B\\ \; & \leq \xi -\phi \left[H+I^{L}+I^{A}+\frac{1}{\tau }\left(Y^{L}+Y^{A}\right)+\frac{\gamma }{2\kappa }P+\frac{\pi \gamma }{2\eta \kappa }B\right]=\xi -\phi \Psi , \end{array}$$

where $\phi =\min\{\alpha ,\theta ,\frac{\gamma }{2},\varpi ,\varphi ,\beta ,\lambda \}$. This implies that

$$\lim_{t\to \infty }\sup\Psi \left(t\right)\leq \frac{\xi }{\phi }=\Omega_{2}.$$

It follows that

$$\begin{array}{cc} \lim_{t\to \infty }\sup I^{L}\left(t\right) & \leq \Omega_{2},\;\lim_{t\to \infty }\sup I^{A}\left(t\right)\leq \Omega_{2},\;\lim_{t\to \infty }\sup Y^{L}\left(t\right)\leq \Omega_{3},\\ \lim_{t\to \infty }\sup Y^{A}\left(t\right) & \leq \Omega_{3},\;\lim_{t\to \infty }\sup P\left(t\right)\leq \Omega_{4},\;\lim_{t\to \infty }\sup B\left(t\right)\leq \Omega_{5}, \end{array}$$

where $\Omega_{3}=\tau \Omega_{2}$, $\Omega_{4}=\frac{2\kappa \Omega_{2}}{\gamma }$ and $\Omega_{5}=\frac{2\eta \kappa \Omega_{2}}{\pi \gamma }$.

It can be verified that the compact set

$$\begin{array}{cc} \Theta & =\left.\left\{\left(H,I^{L},I^{A},Y^{L},Y^{A},P,B\right)\in R_{\geq 0}^{7}:H\leq \Omega_{1},\right.\right.\\ \; & \;\;\;\;\;\;\;\left.\left.H+I^{L}+I^{A}+\frac{1}{\tau }\left(Y^{L}+Y^{A}\right)+\frac{\gamma }{2\kappa }P+\frac{\pi \gamma }{2\eta \kappa }B\leq \Omega_{2}\right\}\right. \end{array}$$

is positively invariant for system (4). □

### 2.2. Steady States and Threshold Parameters

In this section, we find all steady states of the model and establish their existence in terms of four threshold parameters. To calculate the steady states of model (4), we solve

$$\begin{array}{cc} 0 & =\xi -\alpha H-\psi_{1}HP-\psi_{2}HI^{A}-\psi_{3}HY^{A},\\ 0 & =\left(1-\delta \right)\left(\psi_{1}HP+\psi_{2}HI^{A}\right)-\left(\varepsilon +\theta \right)I^{L},\\ 0 & =\delta \left(\psi_{1}HP+\psi_{2}HI^{A}\right)+\varepsilon I^{L}-\gamma I^{A},\\ 0 & =\tau \psi_{3}HY^{A}-\left(\rho +\varpi \right)Y^{L},\\ 0 & =\rho Y^{L}-\varphi Y^{A},\\ 0 & =\kappa I^{A}-\beta P-\pi BP,\\ 0 & =\left(\eta P-\lambda \right)B. \end{array}$$

We find that system (4) has five steady states:

(i) The infection-free steady state, ${\bar{\Delta }}_{0}=\left({\bar{H}}_{0},0,0,0,0,0,0\right)$, where ${\bar{H}}_{0}=\xi /\alpha$. This steady state describes the case of a healthy state where both HIV-1 and HTLV-I are cleared out from the body.

(ii) The infected HIV-1 mono-infection steady state with inefficacious humoral immunity, ${\bar{\Delta }}_{1}=\left({\bar{H}}_{1},{\bar{I}}_{1}^{L},{\bar{I}}_{1}^{A},0,0,{\bar{P}}_{1},0\right),$ where

(6) $$\begin{array}{cc} {\bar{H}}_{1} & =\frac{{\bar{H}}_{0}}{{\bar{ℜ}}_{1}},\;{\bar{I}}_{1}^{L}=\frac{\gamma \beta \alpha \left(1-\delta \right)}{\left(\kappa \psi_{1}+\beta \psi_{2}\right)\left(\delta \theta +\varepsilon \right)}\left({\bar{ℜ}}_{1}-1\right),\\ {\bar{I}}_{1}^{A} & =\frac{\beta \alpha }{\kappa \psi_{1}+\beta \psi_{2}}\left({\bar{ℜ}}_{1}-1\right),\;{\bar{P}}_{1}=\frac{\kappa \alpha }{\kappa \psi_{1}+\beta \psi_{2}}\left({\bar{ℜ}}_{1}-1\right), \end{array}$$

and

$${\bar{ℜ}}_{1}=\frac{{\bar{H}}_{0}\left(\kappa \psi_{1}+\beta \psi_{2}\right)\left(\delta \theta +\varepsilon \right)}{\gamma \beta \left(\theta +\varepsilon \right)}={\bar{ℜ}}_{11}+{\bar{ℜ}}_{12},$$

where

$${\bar{ℜ}}_{11}=\frac{{\bar{H}}_{0}\kappa \psi_{1}\left(\delta \theta +\varepsilon \right)}{\gamma \beta \left(\theta +\varepsilon \right)},\;\;\;\;\;\;\;{\bar{ℜ}}_{12}=\frac{{\bar{H}}_{0}\psi_{2}\left(\delta \theta +\varepsilon \right)}{\gamma \left(\theta +\varepsilon \right)}.$$

${\bar{ℜ}}_{1}$ denotes the basic HIV-1 mono-infection reproductive ratio for system (4). Precisely, ${\bar{ℜ}}_{11}$ and ${\bar{ℜ}}_{12}$ refer to the basic HIV-1 mono-infection reproductive ratios corresponding to V-T-C and C-T-C infections, respectively.

(iii) The infected HTLV-I mono-infection steady state, ${\bar{\Delta }}_{2}=\left({\bar{H}}_{2},0,0,{\bar{Y}}_{2}^{L},{\bar{Y}}_{2}^{A},0,0\right)$, where

$${\bar{H}}_{2}=\frac{{\bar{H}}_{0}}{{\bar{ℜ}}_{2}},\;\;\;{\bar{Y}}_{2}^{L}=\frac{\alpha \varphi }{\psi_{3}\rho }\left({\bar{ℜ}}_{2}-1\right),\;\;\;{\bar{Y}}_{2}^{A}=\frac{\alpha }{\psi_{3}}\left({\bar{ℜ}}_{2}-1\right),$$

and ${\bar{ℜ}}_{2}$ is the basic HTLV-I mono-infection reproductive ratio for system (4) and is defined as:

$${\bar{ℜ}}_{2}=\frac{\tau \psi_{3}\rho {\bar{H}}_{0}}{\varphi \left(\rho +\varpi \right)}.$$

(iv) The infected HIV-1 mono-infection steady state with efficacious humoral immunity, ${\bar{\Delta }}_{3}=\left({\bar{H}}_{3},{\bar{I}}_{3}^{L},{\bar{I}}_{3}^{A},0,0,{\bar{P}}_{3},{\bar{B}}_{3}\right)$, where

(7) $$\begin{array}{cc} {\bar{H}}_{3} & =\frac{\xi \eta }{\psi_{1}\lambda +\alpha \eta +\psi_{2}\eta {\bar{I}}_{3}^{A}},\;\;\;{\bar{I}}_{3}^{L}=\frac{\gamma \left(1-\delta \right)}{\delta \left(\varepsilon +\theta \right)+\varepsilon }{\bar{I}}_{3}^{A},\\ {\bar{P}}_{3} & =\frac{\lambda }{\eta },\;\;\;{\bar{B}}_{3}=\frac{\beta }{\pi }\left(\frac{\eta \kappa {\bar{I}}_{3}^{A}}{\beta \lambda }-1\right), \end{array}$$

and ${\bar{I}}_{3}^{A}$ satisfies the quadratic equation

(8) $${\bar{\varkappa }}_{1}{\left({\bar{I}}_{3}^{A}\right)}^{2}+{\bar{\varkappa }}_{2}{\bar{I}}_{3}^{A}+{\bar{\varkappa }}_{3}=0,$$

where

$${\bar{\varkappa }}_{1}=\gamma \eta \psi_{2}\left(\theta +\varepsilon \right),\;\;\;\;{\bar{\varkappa }}_{2}=\gamma \left(\theta +\varepsilon \right)\left(\lambda \psi_{1}+\alpha \eta \right)-\psi_{2}\xi \eta \left(\delta \theta +\varepsilon \right),\;\;\;\;{\bar{\varkappa }}_{3}=-\xi \lambda \psi_{1}\left(\delta \theta +\varepsilon \right).$$

Since ${\bar{\varkappa }}_{1}>0$ and ${\bar{\varkappa }}_{3}<0$, then ${\bar{\varkappa }}_{2}^{2}-4{\bar{\varkappa }}_{1}{\bar{\varkappa }}_{3}>0$ and Equation (8) has a positive root

$${\bar{I}}_{3}^{A}=\frac{-{\bar{\varkappa }}_{2}+\sqrt{{\bar{\varkappa }}_{2}^{2}-4{\bar{\varkappa }}_{1}{\bar{\varkappa }}_{3}}}{2{\bar{\varkappa }}_{1}}.$$

It follows that ${\bar{H}}_{3}>0,$ ${\bar{I}}_{3}^{L}>0$ and ${\bar{B}}_{3}>0$ only when $\frac{\eta \kappa {\bar{I}}_{3}^{A}}{\beta \lambda }>1$. The HIV-1-specific humoral immunity reproductive ratio in the case of HIV-1 mono-infection is given as:

$${\bar{ℜ}}_{3}=\frac{\eta \kappa {\bar{I}}_{3}^{A}}{\beta \lambda }.$$

Thus, ${\bar{B}}_{3}=\frac{\beta }{\pi }\left({\bar{ℜ}}_{3}-1\right)$.

(v) The infected HIV-1/HTLV-I co-infection steady state with efficacious humoral immunity, ${\bar{\Delta }}_{4}=\left({\bar{H}}_{4},{\bar{I}}_{4}^{L},{\bar{I}}_{4}^{A},{\bar{Y}}_{4}^{L},{\bar{Y}}_{4}^{A},{\bar{P}}_{4},{\bar{B}}_{4}\right)$, where

$$\begin{array}{cc} {\bar{H}}_{4} & =\frac{\varphi \left(\rho +\varpi \right)}{\tau \psi_{3}\rho }={\bar{H}}_{2},\;\;\;\;{\bar{I}}_{4}^{L}=\frac{\gamma \psi_{1}\lambda \left(1-\delta \right)}{\eta \psi_{2}\left(\delta \theta +\varepsilon \right)\left({\bar{ℜ}}_{4}^{*}-1\right)},\\ {\bar{I}}_{4}^{A} & =\frac{\psi_{1}\lambda }{\eta \psi_{2}\left({\bar{ℜ}}_{4}^{*}-1\right)},\;\;\;\;{\bar{P}}_{4}=\frac{\lambda }{\eta }={\bar{P}}_{3},\\ {\bar{Y}}_{4}^{L} & =\frac{\xi \tau }{\rho +\varpi }\left(\frac{{\bar{ℜ}}_{4}-1}{{\bar{ℜ}}_{4}}\right),\;\;\;\;\;{\bar{Y}}_{4}^{A}=\frac{\xi \tau \rho }{\varphi \left(\rho +\varpi \right)}\left(\frac{{\bar{ℜ}}_{4}-1}{{\bar{ℜ}}_{4}}\right),\\ {\bar{B}}_{4} & =\frac{\gamma \beta \psi_{3}\tau \rho \left(\theta +\varepsilon \right)}{\pi \varphi \psi_{2}\left(\rho +\varpi \right)\left(\delta \theta +\varepsilon \right)\left({\bar{ℜ}}_{4}^{*}-1\right)}\left(\frac{{\bar{ℜ}}_{1}}{{\bar{ℜ}}_{2}}-1\right). \end{array}$$

We note that $\Delta_{4}$ exists when $\frac{{\bar{ℜ}}_{1}}{{\bar{ℜ}}_{2}}>1,$ ${\bar{ℜ}}_{4}^{*}>1$ and ${\bar{ℜ}}_{4}>1.$ The competed HTLV-I reproductive ratio in the case of HIV-1/HTLV-I co-infection is stated as:

$${\bar{ℜ}}_{4}=\frac{\xi \tau \psi_{3}\rho \eta \psi_{2}\left(\delta \theta +\varepsilon \right)\left({\bar{ℜ}}_{4}^{*}-1\right)}{\gamma \psi_{1}\lambda \tau \psi_{3}\rho \left(\theta +\varepsilon \right)+\alpha \eta \varphi \psi_{2}\left(\rho +\varpi \right)\left(\delta \theta +\varepsilon \right)\left({\bar{ℜ}}_{4}^{*}-1\right)},$$

where

$${\bar{ℜ}}_{4}^{*}=\frac{\gamma \psi_{3}\tau \rho \left(\theta +\varepsilon \right)}{\varphi \psi_{2}\left(\rho +\varpi \right)\left(\delta \theta +\varepsilon \right)}.$$

According to the above discussion, we sum up the existence conditions for all steady states in Table 2.

### 2.3. Global Stability

We demonstrate the global asymptotic stability of all steady states in this section by establishing appropriate Lyapunov functions [57,58,59]. Define a function Φ(υ)=υ−1−lnυ.

We will use the following geometric-arithmetic mean inequality:

(9) $$\sqrt[{n}]{\prod_{i=1}^{n}{ϝ}_{i}}\leq \frac{1}{n}\sum_{i=1}^{n}{ϝ}_{i},\;\;\;\;{ϝ}_{i}>0,\;\;i=1,2,\cdots$$

Define function ${\bar{\Gamma }}_{j}\left(H,I^{L},I^{A},Y^{L},Y^{A},P,B\right)$ and let ${\bar{\Pi }}_{j}^{{}^{\prime }}$ be the largest invariant subset of

$${\bar{\Pi }}_{j}=\left\{\left(H,I^{L},I^{A},Y^{L},Y^{A},P,B\right):\frac{d{\bar{\Gamma }}_{j}}{dt}=0\right\},\;\;\;j=0,1,\cdots ,4.$$

**Theorem** **1.** *(a) Assume that ${\bar{ℜ}}_{1}\leq 1$ and ${\bar{ℜ}}_{2}\leq 1$; then ${\bar{\Delta }}_{0}$ is globally asymptotically stable (GAS). (b) If ${\bar{ℜ}}_{1}>1$ or ${\bar{ℜ}}_{2}>1$, then ${\bar{\Delta }}_{0}$ is unstable.*

**Proof.**  (a) Construct a function ${\bar{\Gamma }}_{0}\left(H,I^{L},I^{A},Y^{L},Y^{A},P,B\right)$ as:

$${\bar{\Gamma }}_{0}={\bar{H}}_{0}\Phi \left(\frac{H}{{\bar{H}}_{0}}\right)+\frac{\varepsilon }{\delta \theta +\varepsilon }I^{L}+\frac{\theta +\varepsilon }{\delta \theta +\varepsilon }I^{A}+\frac{1}{\tau }Y^{L}+\frac{\rho +\varpi }{\tau \rho }Y^{A}+\frac{\psi_{1}{\bar{H}}_{0}}{\beta }P+\frac{\pi \psi_{1}{\bar{H}}_{0}}{\eta \beta }B.$$

We calculate $\frac{d{\bar{\Gamma }}_{0}}{dt}$ as:

$$\begin{array}{cc} \frac{d{\bar{\Gamma }}_{0}}{dt} & =\left(1-\frac{{\bar{H}}_{0}}{H}\right)\left[\xi -\alpha H-\psi_{1}HP-\psi_{2}HI^{A}-\psi_{3}HY^{A}\right]+\frac{\varepsilon }{\delta \theta +\varepsilon }\left[\left(1-\delta \right)\left(\psi_{1}HP+\psi_{2}HI^{A}\right)-\left(\varepsilon +\theta \right)I^{L}\right]\\ \; & +\frac{\theta +\varepsilon }{\delta \theta +\varepsilon }\left[\delta \left(\psi_{1}HP+\psi_{2}HI^{A}\right)+\varepsilon I^{L}-\gamma I^{A}\right]+\frac{1}{\tau }\left[\tau \psi_{3}HY^{A}-\left(\rho +\varpi \right)Y^{L}\right]+\frac{\rho +\varpi }{\tau \rho }\left[\rho Y^{L}-\varphi Y^{A}\right]\\ \; & +\frac{\psi_{1}{\bar{H}}_{0}}{\beta }\left[\kappa I^{A}-\beta P-\pi BP\right]+\frac{\pi \psi_{1}{\bar{H}}_{0}}{\eta \beta }\left[\eta BP-\lambda B\right]\\ \; & =\left(1-\frac{{\bar{H}}_{0}}{H}\right)\left(\xi -\alpha H\right)+\psi_{2}{\bar{H}}_{0}I^{A}+\psi_{3}{\bar{H}}_{0}Y^{A}-\frac{\gamma \left(\theta +\varepsilon \right)}{\delta \theta +\varepsilon }I^{A}-\frac{\varphi \left(\rho +\varpi \right)}{\tau \rho }Y^{A}+\frac{\kappa \psi_{1}{\bar{H}}_{0}}{\beta }I^{A}-\frac{\pi \lambda \psi_{1}{\bar{H}}_{0}}{\eta \beta }B. \end{array}$$

Using ${\bar{H}}_{0}=\xi /\alpha$, we obtain

$$\frac{d{\bar{\Gamma }}_{0}}{dt}=-\alpha \frac{{\left(H-{\bar{H}}_{0}\right)}^{2}}{H}+\frac{\gamma \left(\theta +\varepsilon \right)}{\delta \theta +\varepsilon }\left({\bar{ℜ}}_{1}-1\right)I^{A}+\frac{\varphi \left(\rho +\varpi \right)}{\tau \rho }\left({\bar{ℜ}}_{2}-1\right)Y^{A}-\frac{\pi \lambda \psi_{1}{\bar{H}}_{0}}{\eta \beta }B.$$

Therefore, $\frac{d{\bar{\Gamma }}_{0}}{dt}\leq 0$ in Θ and $\frac{d{\bar{\Gamma }}_{0}}{dt}=0$ when $H={\bar{H}}_{0}$ and $I^{A}=Y^{A}=B=0.$ The solutions of system (4) converge to the invariant set ${\bar{\Pi }}_{0}^{{}^{\prime }}$. The elements of ${\bar{\Pi }}_{0}^{{}^{\prime }}$ satisfy $H\left(t\right)={\bar{H}}_{0}$ and $Y^{A}\left(t\right)=I^{A}\left(t\right)=0$ and then, $\frac{dH(t)}{dt}=\frac{dY^{A}\left(t\right)}{dt}=0$. From the first and fifth equations of system (4) we have

$$\begin{array}{cc} 0 & =\frac{dH(t)}{dt}=\xi -\alpha {\bar{H}}_{0}-\psi_{1}{\bar{H}}_{0}P\left(t\right)⟹P\left(t\right)=0,\\ 0 & =\frac{dY^{A}\left(t\right)}{dt}=\rho Y^{L}\left(t\right)⟹Y^{L}\left(t\right)=0. \end{array}$$

Furthermore, we have $\frac{dI^{A}\left(t\right)}{dt}=0$ and, from the third equation of system (4), we obtain

$$0=\frac{dI^{A}\left(t\right)}{dt}=\varepsilon I^{L}\left(t\right),$$

which indicates that $I^{L}\left(t\right)=0$ for all *t*. Therefore, ${\bar{\Pi }}_{0}^{{}^{\prime }}=\left\{{\bar{\Delta }}_{0}\right\}$ and, applying the Lyapunov–LaSalle asymptotic stability theorem [60,61,62], we can observe that ${\bar{\Delta }}_{0}$ is GAS.

To prove (b), we need to find the characteristic equation atthe steady state. We calculate the Jacobian matrix $J=J(H,I^{L},I^{A},Y^{L},Y^{A},P,B)$ of system (4) in the following form:

(10) $$J=\left(\begin{array}{ccccccc} -\left(\alpha +\psi_{1}P+\psi_{2}I^{A}+\psi_{3}Y^{A}\right) & 0 & -\psi_{2}H & 0 & -\psi_{3}H & -\psi_{1}H & 0\\ \left(1-\delta \right)\left(\psi_{1}P+\psi_{2}I^{A}\right) & -\left(\varepsilon +\theta \right) & \left(1-\delta \right)\psi_{2}H & 0 & 0 & \left(1-\delta \right)\psi_{1}H & 0\\ \delta \left(\psi_{1}P+\psi_{2}I^{A}\right) & \varepsilon & \delta \psi_{2}H-\gamma & 0 & 0 & \delta \psi_{1}H & 0\\ \tau \psi_{3}Y^{A} & 0 & 0 & -\left(\rho +\varpi \right) & \tau \psi_{3}H & 0 & 0\\ 0 & 0 & 0 & \rho & -\varphi & 0 & 0\\ 0 & 0 & \kappa & 0 & 0 & -\left(\beta +\pi B\right) & -\pi P\\ 0 & 0 & 0 & 0 & 0 & \eta B & \eta P-\lambda \end{array}\right).$$

Then, the characteristic equation at the steady state ${\bar{\Delta }}_{0}$ is given by

$$\begin{array}{cc} \; & \left.\det(J-\Delta I)=(\Delta +\alpha )(\Delta +\lambda )\right.\\ \; & \left.\times \alpha \left(\Delta +\beta \right)\left(\Delta +\gamma \right)\left(\Delta +\varepsilon +\theta \right)-\xi \left(\varepsilon +\delta \left(\Delta +\theta \right)\right)\left(\kappa \psi_{1}+\psi_{2}\left(\Delta +\beta \right)\right)F_{0}\left(\Delta \right)=0,\right. \end{array}$$

where Δ is the eigenvalue and

(11) $$F_{0}\left(\Delta \right)=\alpha \Delta^{2}+\alpha \left(\rho +\varphi +\omega \right)\Delta +\alpha \varphi \left(\rho +\omega \right)\left(1-{\bar{ℜ}}_{2}\right)=0.$$

Clearly, if ${\bar{ℜ}}_{2}>1$, then Equation (11) has a positive root and hence ${\bar{\Delta }}_{0}$ is unstable. □

**Theorem** **2.** *Let ${\bar{ℜ}}_{1}>1,$ $\frac{{\bar{ℜ}}_{2}}{{\bar{ℜ}}_{1}}\leq 1$ and ${\bar{ℜ}}_{3}\leq 1$, then ${\bar{\Delta }}_{1}$ is GAS.*

**Proof.**  Consider a function ${\bar{\Gamma }}_{1}\left(H,I^{L},I^{A},Y^{L},Y^{A},P,B\right)$ as:

$$\begin{array}{cc} {\bar{\Gamma }}_{1} & ={\bar{H}}_{1}\Phi \left(\frac{H}{{\bar{H}}_{1}}\right)+\frac{\varepsilon }{\delta \theta +\varepsilon }{\bar{I}}_{1}^{L}\Phi \left(\frac{I^{L}}{{\bar{I}}_{1}^{L}}\right)+\frac{\theta +\varepsilon }{\delta \theta +\varepsilon }{\bar{I}}_{1}^{A}\Phi \left(\frac{I^{A}}{{\bar{I}}_{1}^{A}}\right)\\ \; & +\frac{1}{\tau }Y^{L}+\frac{\rho +\varpi }{\tau \rho }Y^{A}+\frac{\psi_{1}{\bar{H}}_{1}}{\beta }{\bar{P}}_{1}\Phi \left(\frac{P}{{\bar{P}}_{1}}\right)+\frac{\pi \psi_{1}{\bar{H}}_{1}}{\eta \beta }B. \end{array}$$

Calculating $\frac{d{\bar{\Gamma }}_{1}}{dt}$ as:

$$\begin{array}{cc} \frac{d{\bar{\Gamma }}_{1}}{dt} & =\left(1-\frac{{\bar{H}}_{1}}{H}\right)\left[\xi -\alpha H-\psi_{1}HP-\psi_{2}HI^{A}-\psi_{3}HY^{A}\right]\\ \; & +\frac{\varepsilon }{\delta \theta +\varepsilon }\left(1-\frac{{\bar{I}}_{1}^{L}}{I^{L}}\right)\left[\left(1-\delta \right)\left(\psi_{1}HP+\psi_{2}HI^{A}\right)-\left(\varepsilon +\theta \right)I^{L}\right]\\ \; & +\frac{\theta +\varepsilon }{\delta \theta +\varepsilon }\left(1-\frac{{\bar{I}}_{1}^{A}}{I^{A}}\right)\left[\delta \left(\psi_{1}HP+\psi_{2}HI^{A}\right)+\varepsilon I^{L}-\gamma I^{A}\right]\\ \; & +\frac{1}{\tau }\left[\tau \psi_{3}HY^{A}-\left(\rho +\varpi \right)Y^{L}\right]+\frac{\rho +\varpi }{\tau \rho }\left[\rho Y^{L}-\varphi Y^{A}\right]\\ \; & +\frac{\psi_{1}{\bar{H}}_{1}}{\beta }\left(1-\frac{{\bar{P}}_{1}}{P}\right)\left[\kappa I^{A}-\beta P-\pi BP\right]+\frac{\pi \psi_{1}{\bar{H}}_{1}}{\eta \beta }\left[\eta BP-\lambda B\right]\\ \; & =\left(1-\frac{{\bar{H}}_{1}}{H}\right)\left(\xi -\alpha H\right)+\psi_{2}{\bar{H}}_{1}I^{A}+\psi_{3}{\bar{H}}_{1}Y^{A}-\frac{\varepsilon \left(1-\delta \right)}{\delta \theta +\varepsilon }\left(\psi_{1}HP+\psi_{2}HI^{A}\right)\frac{{\bar{I}}_{1}^{L}}{I^{L}}+\frac{\varepsilon \left(\theta +\varepsilon \right)}{\delta \theta +\varepsilon }{\bar{I}}_{1}^{L}\\ \; & -\frac{\gamma \left(\theta +\varepsilon \right)}{\delta \theta +\varepsilon }I^{A}-\frac{\delta \left(\theta +\varepsilon \right)}{\delta \theta +\varepsilon }\psi_{1}HP\frac{{\bar{I}}_{1}^{A}}{I^{A}}-\frac{\delta \left(\theta +\varepsilon \right)}{\delta \theta +\varepsilon }\psi_{2}H{\bar{I}}_{1}^{A}-\frac{\varepsilon \left(\theta +\varepsilon \right)}{\delta \theta +\varepsilon }I^{L}\frac{{\bar{I}}_{1}^{A}}{I^{A}}+\frac{\gamma \left(\theta +\varepsilon \right)}{\delta \theta +\varepsilon }{\bar{I}}_{1}^{A}\\ \; & -\frac{\varphi \left(\rho +\varpi \right)}{\tau \rho }Y^{A}+\frac{\kappa \psi_{1}{\bar{H}}_{1}}{\beta }I^{A}-\frac{\kappa \psi_{1}{\bar{H}}_{1}}{\beta }I^{A}\frac{{\bar{P}}_{1}}{P}+\psi_{1}{\bar{H}}_{1}{\bar{P}}_{1}+\frac{\pi \psi_{1}{\bar{H}}_{1}}{\beta }B{\bar{P}}_{1}-\frac{\pi \lambda \psi_{1}{\bar{H}}_{1}}{\eta \beta }B. \end{array}$$

Using the steady state conditions for ${\bar{\Delta }}_{1}$, we obtain

$$\begin{array}{cc} \xi & =\alpha {\bar{H}}_{1}+\psi_{1}{\bar{H}}_{1}{\bar{P}}_{1}+\psi_{2}{\bar{H}}_{1}{\bar{I}}_{1}^{A},\;\;\;\;\frac{\varepsilon \left(1-\delta \right)}{\delta \theta +\varepsilon }\left(\psi_{1}{\bar{H}}_{1}{\bar{P}}_{1}+\psi_{2}{\bar{H}}_{1}{\bar{I}}_{1}^{A}\right)=\frac{\varepsilon \left(\theta +\varepsilon \right)}{\delta \theta +\varepsilon }{\bar{I}}_{1}^{L},\\ \; & \left.\psi_{1}{\bar{H}}_{1}{\bar{P}}_{1}+\psi_{2}{\bar{H}}_{1}{\bar{I}}_{1}^{A}=\frac{\gamma \left(\theta +\varepsilon \right)}{\delta \theta +\varepsilon }{\bar{I}}_{1}^{A},\;\;\;\;{\bar{P}}_{1}=\frac{\kappa {\bar{I}}_{1}^{A}}{\beta }.\right. \end{array}$$

Then, we obtain

(12) $$\begin{array}{cc} \frac{d{\bar{\Gamma }}_{1}}{dt} & =\left(1-\frac{{\bar{H}}_{1}}{H}\right)\left(\alpha {\bar{H}}_{1}-\alpha H\right)+\left(\psi_{1}{\bar{H}}_{1}{\bar{P}}_{1}+\psi_{2}{\bar{H}}_{1}{\bar{I}}_{1}^{A}\right)\left(1-\frac{{\bar{H}}_{1}}{H}\right)+\psi_{3}{\bar{H}}_{1}Y^{A}-\frac{\varepsilon \left(1-\delta \right)}{\delta \theta +\varepsilon }\psi_{1}{\bar{H}}_{1}{\bar{P}}_{1}\frac{HP{\bar{I}}_{1}^{L}}{{\bar{H}}_{1}{\bar{P}}_{1}I^{L}}\\ \; & -\frac{\varepsilon \left(1-\delta \right)}{\delta \theta +\varepsilon }\psi_{2}{\bar{H}}_{1}{\bar{I}}_{1}^{A}\frac{HI^{A}{\bar{I}}_{1}^{L}}{{\bar{H}}_{1}{\bar{I}}_{1}^{A}I^{L}}+\frac{\varepsilon \left(1-\delta \right)}{\delta \theta +\varepsilon }\left(\psi_{1}{\bar{H}}_{1}{\bar{P}}_{1}+\psi_{2}{\bar{H}}_{1}{\bar{I}}_{1}^{A}\right)-\frac{\delta \left(\theta +\varepsilon \right)}{\delta \theta +\varepsilon }\psi_{1}{\bar{H}}_{1}{\bar{P}}_{1}\frac{HP{\bar{I}}_{1}^{A}}{{\bar{H}}_{1}{\bar{P}}_{1}I^{A}}\\ \; & -\frac{\delta \left(\theta +\varepsilon \right)}{\delta \theta +\varepsilon }\psi_{2}{\bar{H}}_{1}{\bar{I}}_{1}^{A}\frac{H}{{\bar{H}}_{1}}-\frac{\varepsilon \left(1-\delta \right)}{\delta \theta +\varepsilon }\left(\psi_{1}{\bar{H}}_{1}{\bar{P}}_{1}+\psi_{2}{\bar{H}}_{1}{\bar{I}}_{1}^{A}\right)\frac{I^{L}{\bar{I}}_{1}^{A}}{{\bar{I}}_{1}^{L}I^{A}}+\psi_{1}{\bar{H}}_{1}{\bar{P}}_{1}+\psi_{2}{\bar{H}}_{1}{\bar{I}}_{1}^{A}-\frac{\varphi \left(\rho +\varpi \right)}{\tau \rho }Y^{A}\\ \; & -\psi_{1}{\bar{H}}_{1}{\bar{P}}_{1}\frac{I^{A}{\bar{P}}_{1}}{{\bar{I}}_{1}^{A}P}+\psi_{1}{\bar{H}}_{1}{\bar{P}}_{1}+\frac{\pi \psi_{1}{\bar{H}}_{1}}{\beta }B{\bar{P}}_{1}-\frac{\pi \lambda \psi_{1}{\bar{H}}_{1}}{\eta \beta }B\\ \; & =-\alpha \frac{{\left(H-{\bar{H}}_{1}\right)}^{2}}{H}+\frac{\varepsilon \left(1-\delta \right)}{\delta \theta +\varepsilon }\psi_{1}{\bar{H}}_{1}{\bar{P}}_{1}\left(4-\frac{{\bar{H}}_{1}}{H}-\frac{HP{\bar{I}}_{1}^{L}}{{\bar{H}}_{1}{\bar{P}}_{1}I^{L}}-\frac{I^{L}{\bar{I}}_{1}^{A}}{{\bar{I}}_{1}^{L}I^{A}}-\frac{I^{A}{\bar{P}}_{1}}{{\bar{I}}_{1}^{A}P}\right)\\ \; & +\frac{\varepsilon \left(1-\delta \right)}{\delta \theta +\varepsilon }\psi_{2}{\bar{H}}_{1}{\bar{I}}_{1}^{A}\left(3-\frac{{\bar{H}}_{1}}{H}-\frac{HI^{A}{\bar{I}}_{1}^{L}}{{\bar{H}}_{1}{\bar{I}}_{1}^{A}I^{L}}-\frac{I^{L}{\bar{I}}_{1}^{A}}{{\bar{I}}_{1}^{L}I^{A}}\right)+\frac{\delta \left(\theta +\varepsilon \right)}{\delta \theta +\varepsilon }\psi_{1}{\bar{H}}_{1}{\bar{P}}_{1}\left(3-\frac{{\bar{H}}_{1}}{H}-\frac{HP{\bar{I}}_{1}^{A}}{{\bar{H}}_{1}{\bar{P}}_{1}I^{A}}-\frac{I^{A}{\bar{P}}_{1}}{{\bar{I}}_{1}^{A}P}\right)\\ \; & +\frac{\delta \left(\theta +\varepsilon \right)}{\delta \theta +\varepsilon }\psi_{2}{\bar{H}}_{1}{\bar{I}}_{1}^{A}\left(2-\frac{{\bar{H}}_{1}}{H}-\frac{H}{{\bar{H}}_{1}}\right)+\frac{\varphi \left(\rho +\varpi \right)}{\tau \rho }\left(\frac{\tau \psi_{3}\rho {\bar{H}}_{1}}{\varphi \left(\rho +\varpi \right)}-1\right)Y^{A}+\frac{\pi \psi_{1}{\bar{H}}_{1}}{\beta }\left({\bar{P}}_{1}-\frac{\lambda }{\eta }\right)B. \end{array}$$

Therefore, Equation (12) becomes

(13) $$\begin{array}{cc} \frac{d{\bar{\Gamma }}_{1}}{dt} & =-\left[\alpha +\frac{\delta \psi_{2}{\bar{I}}_{1}^{A}\left(\theta +\varepsilon \right)}{\delta \theta +\varepsilon }\right]\frac{{\left(H-{\bar{H}}_{1}\right)}^{2}}{H}+\frac{\varepsilon \left(1-\delta \right)}{\delta \theta +\varepsilon }\psi_{1}{\bar{H}}_{1}{\bar{P}}_{1}\left(4-\frac{{\bar{H}}_{1}}{H}-\frac{HP{\bar{I}}_{1}^{L}}{{\bar{H}}_{1}{\bar{P}}_{1}I^{L}}-\frac{I^{L}{\bar{I}}_{1}^{A}}{{\bar{I}}_{1}^{L}I^{A}}-\frac{I^{A}{\bar{P}}_{1}}{{\bar{I}}_{1}^{A}P}\right)\\ \; & +\frac{\varepsilon \left(1-\delta \right)}{\delta \theta +\varepsilon }\psi_{2}{\bar{H}}_{1}{\bar{I}}_{1}^{A}\left(3-\frac{{\bar{H}}_{1}}{H}-\frac{HI^{A}{\bar{I}}_{1}^{L}}{{\bar{H}}_{1}{\bar{I}}_{1}^{A}I^{L}}-\frac{I^{L}{\bar{I}}_{1}^{A}}{{\bar{I}}_{1}^{L}I^{A}}\right)+\frac{\delta \left(\theta +\varepsilon \right)}{\delta \theta +\varepsilon }\psi_{1}{\bar{H}}_{1}{\bar{P}}_{1}\left(3-\frac{{\bar{H}}_{1}}{H}-\frac{HP{\bar{I}}_{1}^{A}}{{\bar{H}}_{1}{\bar{P}}_{1}I^{A}}-\frac{I^{A}{\bar{P}}_{1}}{{\bar{I}}_{1}^{A}P}\right)\\ \; & +\frac{\varphi \left(\rho +\varpi \right)}{\tau \rho }\left(\frac{{\bar{ℜ}}_{2}}{{\bar{ℜ}}_{1}}-1\right)Y^{A}+\frac{\pi \psi_{1}{\bar{H}}_{1}}{\beta }\left({\bar{P}}_{1}-{\bar{P}}_{3}\right)B. \end{array}$$

Inequality (9) implies that

$$\begin{array}{cc} \frac{{\bar{H}}_{1}}{H}+\frac{HP{\bar{I}}_{1}^{L}}{{\bar{H}}_{1}{\bar{P}}_{1}I^{L}}+\frac{I^{L}{\bar{I}}_{1}^{A}}{{\bar{I}}_{1}^{L}I^{A}}+\frac{I^{A}{\bar{P}}_{1}}{{\bar{I}}_{1}^{A}P} & \geq 4,\\ \frac{{\bar{H}}_{1}}{H}+\frac{HI^{A}{\bar{I}}_{1}^{L}}{{\bar{H}}_{1}{\bar{I}}_{1}^{A}I^{L}}+\frac{I^{L}{\bar{I}}_{1}^{A}}{{\bar{I}}_{1}^{L}I^{A}} & \geq 3,\\ \frac{{\bar{H}}_{1}}{H}+\frac{HP{\bar{I}}_{1}^{A}}{{\bar{H}}_{1}{\bar{P}}_{1}I^{A}}+\frac{I^{A}{\bar{P}}_{1}}{{\bar{I}}_{1}^{A}P} & \geq 3. \end{array}$$

Since ${\bar{ℜ}}_{3}\leq 1$ then ${\bar{\Delta }}_{3}$ does not exist. Thus, $\frac{dB}{dt}=\eta \left(P\left(t\right)-\frac{\lambda }{\eta }\right)B\left(t\right)=\eta \left(P\left(t\right)-{\bar{P}}_{3}\right)B\left(t\right)\leq 0$, and then ${\bar{P}}_{1}\leq {\bar{P}}_{3}$. In addition, since $\frac{{\bar{ℜ}}_{2}}{{\bar{ℜ}}_{1}}\leq 1$, then $\frac{d{\bar{\Gamma }}_{1}}{dt}\leq 0$ in Θ with $\frac{d{\bar{\Gamma }}_{1}}{dt}=0$ occurs when $H={\bar{H}}_{1}$, $I^{L}={\bar{I}}_{1}^{L}$, $I^{A}={\bar{I}}_{1}^{A}$, $P={\bar{P}}_{1}$ and $Y^{A}=B=0.$ The solutions of system (4) tend to the invariant set ${\bar{\Pi }}_{1}^{\prime }$ which has elements satisfying $Y^{A}\left(t\right)=0$. The fifth equation of system (4) implies

$$0=\frac{dY^{A}\left(t\right)}{dt}=\rho Y^{L}\left(t\right)⟹Y^{L}\left(t\right)=0.$$

Hence, ${\bar{\Pi }}_{1}^{\prime }=\left\{\bar{\Delta }{}_{1}\right\}$ and then the Lyapunov–LaSalle asymptotic stability theorem implies that ${\bar{\Delta }}_{1}$ is GAS. □

**Theorem** **3.** *Let ${\bar{ℜ}}_{2}>1$ and $\frac{{\bar{ℜ}}_{1}}{{\bar{ℜ}}_{2}}\leq 1$, then ${\bar{\Delta }}_{2}$ is GAS.*

**Proof.**  Consider a function ${\bar{\Gamma }}_{2}\left(H,I^{L},I^{A},Y^{L},Y^{A},P,B\right)$ as:

$$\begin{array}{cc} {\bar{\Gamma }}_{2} & ={\bar{H}}_{2}\Phi \left(\frac{H}{{\bar{H}}_{2}}\right)+\frac{\varepsilon }{\delta \theta +\varepsilon }I^{L}+\frac{\theta +\varepsilon }{\delta \theta +\varepsilon }I^{A}+\frac{1}{\tau }{\bar{Y}}_{2}^{L}\Phi \left(\frac{Y^{L}}{{\bar{Y}}_{2}^{L}}\right)\\ \; & +\frac{\rho +\varpi }{\tau \rho }{\bar{Y}}_{2}^{A}\Phi \left(\frac{Y^{A}}{{\bar{Y}}_{2}^{A}}\right)+\frac{\psi_{1}{\bar{H}}_{2}}{\beta }P+\frac{\pi \psi_{1}{\bar{H}}_{2}}{\eta \beta }B. \end{array}$$

We calculate $\frac{d{\bar{\Gamma }}_{2}}{dt}$ as:

$$\begin{array}{cc} \frac{d{\bar{\Gamma }}_{2}}{dt} & =\left(1-\frac{{\bar{H}}_{2}}{H}\right)\left[\xi -\alpha H-\psi_{1}HP-\psi_{2}HI^{A}-\psi_{3}HY^{A}\right]+\frac{\varepsilon }{\delta \theta +\varepsilon }\left[\left(1-\delta \right)\left(\psi_{1}HP+\psi_{2}HI^{A}\right)-\left(\varepsilon +\theta \right)I^{L}\right]\\ \; & +\frac{\theta +\varepsilon }{\delta \theta +\varepsilon }\left[\delta \left(\psi_{1}HP+\psi_{2}HI^{A}\right)+\varepsilon I^{L}-\gamma I^{A}\right]+\frac{1}{\tau }\left(1-\frac{{\bar{Y}}_{2}^{L}}{Y^{L}}\right)\left[\tau \psi_{3}HY^{A}-\left(\rho +\varpi \right)Y^{L}\right]\\ \; & +\frac{\rho +\varpi }{\tau \rho }\left(1-\frac{{\bar{Y}}_{2}^{A}}{Y^{A}}\right)\left[\rho Y^{L}-\varphi Y^{A}\right]+\frac{\psi_{1}{\bar{H}}_{2}}{\beta }\left[\kappa I^{A}-\beta P-\pi BP\right]+\frac{\pi \psi_{1}{\bar{H}}_{2}}{\eta \beta }\left[\eta BP-\lambda B\right]\\ \; & =\left(1-\frac{{\bar{H}}_{2}}{H}\right)\left(\xi -\alpha H\right)+\psi_{2}{\bar{H}}_{2}I^{A}+\psi_{3}{\bar{H}}_{2}Y^{A}-\frac{\gamma \left(\theta +\varepsilon \right)}{\delta \theta +\varepsilon }I^{A}-\psi_{3}HY^{A}\frac{{\bar{Y}}_{2}^{L}}{Y^{L}}+\frac{\rho +\varpi }{\tau }{\bar{Y}}_{2}^{L}\\ \; & -\frac{\varphi \left(\rho +\varpi \right)}{\tau \rho }Y^{A}-\frac{\rho +\varpi }{\tau }Y^{L}\frac{{\bar{Y}}_{2}^{A}}{Y^{A}}+\frac{\varphi \left(\rho +\varpi \right)}{\tau \rho }{\bar{Y}}_{2}^{A}+\frac{\kappa \psi_{1}{\bar{H}}_{2}}{\beta }I^{A}-\frac{\pi \lambda \psi_{1}{\bar{H}}_{2}}{\eta \beta }B. \end{array}$$

Utilizing the steady state conditions for ${\bar{\Delta }}_{2}$:

$$\xi =\alpha {\bar{H}}_{2}+\psi_{3}{\bar{H}}_{2}{\bar{Y}}_{2}^{A},\;\;\;\;\psi_{3}{\bar{H}}_{2}{\bar{Y}}_{2}^{A}=\frac{\rho +\varpi }{\tau }{\bar{Y}}_{2}^{L}=\frac{\varphi \left(\rho +\varpi \right)}{\tau \rho }{\bar{Y}}_{2}^{A},$$

we obtain

(14) $$\begin{array}{cc} \frac{d{\bar{\Gamma }}_{2}}{dt} & =\left(1-\frac{{\bar{H}}_{2}}{H}\right)\left(\alpha {\bar{H}}_{2}-\alpha H\right)+\psi_{3}{\bar{H}}_{2}{\bar{Y}}_{2}^{A}\left(1-\frac{{\bar{H}}_{2}}{H}\right)+\psi_{2}{\bar{H}}_{2}I^{A}-\frac{\gamma \left(\theta +\varepsilon \right)}{\delta \theta +\varepsilon }I^{A}\\ \; & -\psi_{3}{\bar{H}}_{2}{\bar{Y}}_{2}^{A}\frac{HY^{A}{\bar{Y}}_{2}^{L}}{{\bar{H}}_{2}{\bar{Y}}_{2}^{A}Y^{L}}+\psi_{3}{\bar{H}}_{2}{\bar{Y}}_{2}^{A}-\psi_{3}{\bar{H}}_{2}{\bar{Y}}_{2}^{A}\frac{Y^{L}{\bar{Y}}_{2}^{A}}{{\bar{Y}}_{2}^{L}Y^{A}}+\psi_{3}{\bar{H}}_{2}{\bar{Y}}_{2}^{A}+\frac{\kappa \psi_{1}{\bar{H}}_{2}}{\beta }I^{A}-\frac{\pi \lambda \psi_{1}{\bar{H}}_{2}}{\eta \beta }B\\ \; & =-\alpha \frac{{\left(H-{\bar{H}}_{2}\right)}^{2}}{H}+\psi_{3}{\bar{H}}_{2}{\bar{Y}}_{2}^{A}\left(3-\frac{{\bar{H}}_{2}}{H}-\frac{HY^{A}{\bar{Y}}_{2}^{L}}{{\bar{H}}_{2}{\bar{Y}}_{2}^{A}Y^{L}}-\frac{Y^{L}{\bar{Y}}_{2}^{A}}{{\bar{Y}}_{2}^{L}Y^{A}}\right)\\ \; & +\frac{\gamma \left(\theta +\varepsilon \right)}{\delta \theta +\varepsilon }\left(\frac{{\bar{H}}_{2}\left(\kappa \psi_{1}+\beta \psi_{2}\right)\left(\delta \theta +\varepsilon \right)}{\gamma \beta \left(\theta +\varepsilon \right)}-1\right)I^{A}-\frac{\pi \lambda \psi_{1}{\bar{H}}_{2}}{\eta \beta }B\\ \; & =-\alpha \frac{{\left(H-{\bar{H}}_{2}\right)}^{2}}{H}+\psi_{3}{\bar{H}}_{2}{\bar{Y}}_{2}^{A}\left(3-\frac{{\bar{H}}_{2}}{H}-\frac{HY^{A}{\bar{Y}}_{2}^{L}}{{\bar{H}}_{2}{\bar{Y}}_{2}^{A}Y^{L}}-\frac{Y^{L}{\bar{Y}}_{2}^{A}}{{\bar{Y}}_{2}^{L}Y^{A}}\right)\\ \; & +\frac{\gamma \left(\theta +\varepsilon \right)}{\delta \theta +\varepsilon }\left(\frac{{\bar{ℜ}}_{1}}{{\bar{ℜ}}_{2}}-1\right)I^{A}-\frac{\pi \lambda \psi_{1}{\bar{H}}_{2}}{\eta \beta }B. \end{array}$$

If $\frac{{\bar{ℜ}}_{1}}{{\bar{ℜ}}_{2}}\leq 1$, then, applying inequality (9), we obtain $\frac{d{\bar{\Gamma }}_{2}}{dt}\leq 0$ in Θ with $\frac{d{\bar{\Gamma }}_{2}}{dt}=0$ when $H={\bar{H}}_{2},$ $Y^{L}={\bar{Y}}_{2}^{L},$ $Y^{A}={\bar{Y}}_{2}^{A}$ and $I^{A}=B=0.$ The solutions of system (4) tend to the invariant set ${\bar{\Pi }}_{2}^{{}^{\prime }}$ which contains elements with $H\left(t\right)={\bar{H}}_{2},$ $Y^{A}\left(t\right)={\bar{Y}}_{2}^{A},$ $I^{A}\left(t\right)=0$, then $\frac{dH(t)}{dt}=0$. The first equation of system (4) leads to

$$0=\frac{dH(t)}{dt}=\xi -\alpha {\bar{H}}_{2}-\psi_{1}{\bar{H}}_{2}P\left(t\right)-\psi_{3}{\bar{H}}_{2}{\bar{Y}}_{2}^{A}⟹P\left(t\right)=0.$$

Furthermore, we have $\frac{dI^{A}\left(t\right)}{dt}=0$ and the third equation of system (4) yields

$$0=\frac{dI^{A}\left(t\right)}{dt}=\varepsilon I^{L}\left(t\right)⟹I^{L}\left(t\right)=0.$$

Therefore, ${\bar{\Pi }}_{2}^{{}^{\prime }}=\left\{\bar{\Delta }{}_{2}\right\}$ and ${\bar{\Delta }}_{2}$ is GAS using the Lyapunov–LaSalle asymptotic stability theorem. □

**Theorem** **4.** *If ${\bar{ℜ}}_{3}>1$ and ${\bar{ℜ}}_{4}\leq 1$, then ${\bar{\Delta }}_{3}$ is GAS.*

**Proof.**  Define a function ${\bar{\Gamma }}_{3}\left(H,I^{L},I^{A},Y^{L},Y^{A},P,B\right)$ as:

$$\begin{array}{cc} {\bar{\Gamma }}_{3} & ={\bar{H}}_{3}\Phi \left(\frac{H}{{\bar{H}}_{3}}\right)+\frac{\varepsilon }{\delta \theta +\varepsilon }{\bar{I}}_{3}^{L}\Phi \left(\frac{I^{L}}{{\bar{I}}_{3}^{L}}\right)+\frac{\theta +\varepsilon }{\delta \theta +\varepsilon }{\bar{I}}_{3}^{A}\Phi \left(\frac{I^{A}}{{\bar{I}}_{3}^{A}}\right)+\frac{1}{\tau }Y^{L}\\ \; & +\frac{\rho +\varpi }{\tau \rho }Y^{A}+\frac{\psi_{1}{\bar{H}}_{3}{\bar{P}}_{3}}{\kappa {\bar{I}}_{3}^{A}}{\bar{P}}_{3}\Phi \left(\frac{P}{{\bar{P}}_{3}}\right)+\frac{\pi \psi_{1}{\bar{H}}_{3}{\bar{P}}_{3}}{\eta \kappa {\bar{I}}_{3}^{A}}{\bar{B}}_{3}\Phi \left(\frac{B}{{\bar{B}}_{3}}\right). \end{array}$$

We calculate $\frac{d{\bar{\Gamma }}_{3}}{dt}$ as:

(15) $$\begin{array}{cc} \frac{d{\bar{\Gamma }}_{3}}{dt} & =\left(1-\frac{{\bar{H}}_{3}}{H}\right)\left[\xi -\alpha H-\psi_{1}HP-\psi_{2}HI^{A}-\psi_{3}HY^{A}\right]\\ \; & +\frac{\varepsilon }{\delta \theta +\varepsilon }\left(1-\frac{{\bar{I}}_{3}^{L}}{I^{L}}\right)\left[\left(1-\delta \right)\left(\psi_{1}HP+\psi_{2}HI^{A}\right)-\left(\varepsilon +\theta \right)I^{L}\right]\\ \; & +\frac{\theta +\varepsilon }{\delta \theta +\varepsilon }\left(1-\frac{{\bar{I}}_{3}^{A}}{I^{A}}\right)\left[\delta \left(\psi_{1}HP+\psi_{2}HI^{A}\right)+\varepsilon I^{L}-\gamma I^{A}\right]+\frac{1}{\tau }\left[\tau \psi_{3}HY^{A}-\left(\rho +\varpi \right)Y^{L}\right]\\ \; & +\frac{\rho +\varpi }{\tau \rho }\left[\rho Y^{L}-\varphi Y^{A}\right]+\frac{\psi_{1}{\bar{H}}_{3}{\bar{P}}_{3}}{\kappa {\bar{I}}_{3}^{A}}\left(1-\frac{{\bar{P}}_{3}}{P}\right)\left[\kappa I^{A}-\beta P-\pi BP\right]\\ \; & +\frac{\pi \psi_{1}{\bar{H}}_{3}{\bar{P}}_{3}}{\eta \kappa {\bar{I}}_{3}^{A}}\left(1-\frac{{\bar{B}}_{3}}{B}\right)\left[\eta BP-\lambda B\right]. \end{array}$$

We collect the terms of Equation (15) as:

$$\begin{array}{cc} \frac{d{\bar{\Gamma }}_{3}}{dt} & =\left(1-\frac{{\bar{H}}_{3}}{H}\right)\left(\xi -\alpha H\right)+\psi_{1}{\bar{H}}_{3}P+\psi_{2}{\bar{H}}_{3}I^{A}+\psi_{3}{\bar{H}}_{3}Y^{A}-\frac{\varepsilon \left(1-\delta \right)}{\delta \theta +\varepsilon }\psi_{1}HP\frac{{\bar{I}}_{3}^{L}}{I^{L}}\\ \; & -\frac{\varepsilon \left(1-\delta \right)}{\delta \theta +\varepsilon }\psi_{2}HI^{A}\frac{{\bar{I}}_{3}^{L}}{I^{L}}+\frac{\varepsilon \left(\theta +\varepsilon \right)}{\delta \theta +\varepsilon }{\bar{I}}_{3}^{L}-\frac{\gamma \left(\theta +\varepsilon \right)}{\delta \theta +\varepsilon }I^{A}-\frac{\delta \left(\theta +\varepsilon \right)}{\delta \theta +\varepsilon }\psi_{1}HP\frac{{\bar{I}}_{3}^{A}}{I^{A}}\\ \; & -\frac{\delta \left(\theta +\varepsilon \right)}{\delta \theta +\varepsilon }\psi_{2}H{\bar{I}}_{3}^{A}-\frac{\varepsilon \left(\theta +\varepsilon \right)}{\delta \theta +\varepsilon }I^{L}\frac{{\bar{I}}_{3}^{A}}{I^{A}}+\frac{\gamma \left(\theta +\varepsilon \right)}{\delta \theta +\varepsilon }{\bar{I}}_{3}^{A}-\frac{\varphi \left(\rho +\varpi \right)}{\tau \rho }Y^{A}\\ \; & +\psi_{1}{\bar{H}}_{3}{\bar{P}}_{3}\frac{I^{A}}{{\bar{I}}_{3}^{A}}-\psi_{1}{\bar{H}}_{3}{\bar{P}}_{3}\frac{\beta P}{\kappa {\bar{I}}_{3}^{A}}-\psi_{1}{\bar{H}}_{3}{\bar{P}}_{3}\frac{I^{A}{\bar{P}}_{3}}{{\bar{I}}_{3}^{A}P}+\psi_{1}{\bar{H}}_{3}{\bar{P}}_{3}\frac{\beta {\bar{P}}_{3}}{\kappa {\bar{I}}_{3}^{A}}\\ \; & +\psi_{1}{\bar{H}}_{3}{\bar{P}}_{3}\frac{\pi B{\bar{P}}_{3}}{\kappa {\bar{I}}_{3}^{A}}-\psi_{1}{\bar{H}}_{3}{\bar{P}}_{3}\frac{\pi \lambda B}{\eta \kappa {\bar{I}}_{3}^{A}}-\psi_{1}{\bar{H}}_{3}{\bar{P}}_{3}\frac{\pi {\bar{B}}_{3}P}{\kappa {\bar{I}}_{3}^{A}}+\psi_{1}{\bar{H}}_{3}{\bar{P}}_{3}\frac{\pi \lambda {\bar{B}}_{3}}{\eta \kappa {\bar{I}}_{3}^{A}}. \end{array}$$

The steady state conditions for ${\bar{\Delta }}_{3}$ give:

$$\begin{array}{cc} \xi & =\alpha {\bar{H}}_{3}+\psi_{1}{\bar{H}}_{3}{\bar{P}}_{3}+\psi_{2}{\bar{H}}_{3}{\bar{I}}_{3}^{A},\;\;\;\;\frac{\varepsilon \left(1-\delta \right)}{\delta \theta +\varepsilon }\left(\psi_{1}{\bar{H}}_{3}{\bar{P}}_{3}+\psi_{2}{\bar{H}}_{3}{\bar{I}}_{3}^{A}\right)=\frac{\varepsilon \left(\theta +\varepsilon \right)}{\delta \theta +\varepsilon }{\bar{I}}_{3}^{L},\\ \; & \left.\psi_{1}{\bar{H}}_{3}{\bar{P}}_{3}+\psi_{2}{\bar{H}}_{3}{\bar{I}}_{3}^{A}=\frac{\gamma \left(\theta +\varepsilon \right)}{\delta \theta +\varepsilon }{\bar{I}}_{3}^{A},\;\;\;\kappa {\bar{I}}_{3}^{A}=\beta {\bar{P}}_{3}+\pi {\bar{B}}_{3}{\bar{P}}_{3},\;\;\;\;{\bar{P}}_{3}=\frac{\lambda }{\eta }.\right. \end{array}$$

Then, we obtain

$$\begin{array}{cc} \frac{d{\bar{\Gamma }}_{3}}{dt} & =\left(1-\frac{{\bar{H}}_{3}}{H}\right)\left(\alpha {\bar{H}}_{3}-\alpha H\right)+\left(\psi_{1}{\bar{H}}_{3}{\bar{P}}_{3}+\psi_{2}{\bar{H}}_{3}{\bar{I}}_{3}^{A}\right)\left(1-\frac{{\bar{H}}_{3}}{H}\right)+\left(\psi_{3}{\bar{H}}_{3}-\frac{\varphi \left(\rho +\varpi \right)}{\tau \rho }\right)Y^{A}\\ \; & -\frac{\varepsilon \left(1-\delta \right)}{\delta \theta +\varepsilon }\psi_{1}{\bar{H}}_{3}{\bar{P}}_{3}\frac{HP{\bar{I}}_{3}^{L}}{{\bar{H}}_{3}{\bar{P}}_{3}I^{L}}-\frac{\varepsilon \left(1-\delta \right)}{\delta \theta +\varepsilon }\psi_{2}{\bar{H}}_{3}{\bar{I}}_{3}^{A}\frac{HI^{A}{\bar{I}}_{3}^{L}}{{\bar{H}}_{3}{\bar{I}}_{3}^{A}I^{L}}+\frac{\varepsilon \left(1-\delta \right)}{\delta \theta +\varepsilon }\left(\psi_{1}{\bar{H}}_{3}{\bar{P}}_{3}+\psi_{2}{\bar{H}}_{3}{\bar{I}}_{3}^{A}\right)\\ \; & -\frac{\delta \left(\theta +\varepsilon \right)}{\delta \theta +\varepsilon }\psi_{1}{\bar{H}}_{3}{\bar{P}}_{3}\frac{HP{\bar{I}}_{3}^{A}}{{\bar{H}}_{3}{\bar{P}}_{3}I^{A}}-\frac{\delta \left(\theta +\varepsilon \right)}{\delta \theta +\varepsilon }\psi_{2}{\bar{H}}_{3}{\bar{I}}_{3}^{A}\frac{H}{{\bar{H}}_{3}}-\frac{\varepsilon \left(1-\delta \right)}{\delta \theta +\varepsilon }\left(\psi_{1}{\bar{H}}_{3}{\bar{P}}_{3}+\psi_{2}{\bar{H}}_{3}{\bar{I}}_{3}^{A}\right)\frac{I^{L}{\bar{I}}_{3}^{A}}{{\bar{I}}_{3}^{L}I^{A}}\\ \; & +\psi_{1}{\bar{H}}_{3}{\bar{P}}_{3}+\psi_{2}{\bar{H}}_{3}{\bar{I}}_{3}^{A}-\psi_{1}{\bar{H}}_{3}{\bar{P}}_{3}\frac{I^{A}{\bar{P}}_{3}}{{\bar{I}}_{3}^{A}P}+\psi_{1}{\bar{H}}_{3}{\bar{P}}_{3}\\ \; & =-\alpha \frac{{\left(H-{\bar{H}}_{3}\right)}^{2}}{H}+\frac{\varepsilon \left(1-\delta \right)}{\delta \theta +\varepsilon }\psi_{1}{\bar{H}}_{3}{\bar{P}}_{3}\left(4-\frac{{\bar{H}}_{3}}{H}-\frac{HP{\bar{I}}_{3}^{L}}{{\bar{H}}_{3}{\bar{P}}_{3}I^{L}}-\frac{I^{L}{\bar{I}}_{3}^{A}}{{\bar{I}}_{3}^{L}I^{A}}-\frac{I^{A}{\bar{P}}_{3}}{{\bar{I}}_{3}^{A}P}\right)\\ \; & +\frac{\varepsilon \left(1-\delta \right)}{\delta \theta +\varepsilon }\psi_{2}{\bar{H}}_{3}{\bar{I}}_{3}^{A}\left(3-\frac{{\bar{H}}_{3}}{H}-\frac{HI^{A}{\bar{I}}_{3}^{L}}{{\bar{H}}_{3}{\bar{I}}_{3}^{A}I^{L}}-\frac{I^{L}{\bar{I}}_{3}^{A}}{{\bar{I}}_{3}^{L}I^{A}}\right)+\frac{\delta \left(\theta +\varepsilon \right)}{\delta \theta +\varepsilon }\psi_{1}{\bar{H}}_{3}{\bar{P}}_{3}\left(3-\frac{{\bar{H}}_{3}}{H}-\frac{HP{\bar{I}}_{3}^{A}}{{\bar{H}}_{3}{\bar{P}}_{3}I^{A}}-\frac{I^{A}{\bar{P}}_{3}}{{\bar{I}}_{3}^{A}P}\right)\\ \; & +\frac{\delta \left(\theta +\varepsilon \right)}{\delta \theta +\varepsilon }\psi_{2}{\bar{H}}_{3}{\bar{I}}_{3}^{A}\left(2-\frac{{\bar{H}}_{3}}{H}-\frac{H}{{\bar{H}}_{3}}\right)+\psi_{3}\left({\bar{H}}_{3}-\frac{\varphi \left(\rho +\varpi \right)}{\tau \rho \psi_{3}}\right)Y^{A}\\ \; & =-\left[\alpha +\frac{\delta \psi_{2}{\bar{I}}_{3}^{A}\left(\theta +\varepsilon \right)}{\delta \theta +\varepsilon }\right]\frac{{\left(H-{\bar{H}}_{3}\right)}^{2}}{H}+\frac{\varepsilon \left(1-\delta \right)}{\delta \theta +\varepsilon }\psi_{1}{\bar{H}}_{3}{\bar{P}}_{3}\left(4-\frac{{\bar{H}}_{3}}{H}-\frac{HP{\bar{I}}_{3}^{L}}{{\bar{H}}_{3}{\bar{P}}_{3}I^{L}}-\frac{I^{L}{\bar{I}}_{3}^{A}}{{\bar{I}}_{3}^{L}I^{A}}-\frac{I^{A}{\bar{P}}_{3}}{{\bar{I}}_{3}^{A}P}\right)\\ \; & +\frac{\varepsilon \left(1-\delta \right)}{\delta \theta +\varepsilon }\psi_{2}{\bar{H}}_{3}{\bar{I}}_{3}^{A}\left(3-\frac{{\bar{H}}_{3}}{H}-\frac{HI^{A}{\bar{I}}_{3}^{L}}{{\bar{H}}_{3}{\bar{I}}_{3}^{A}I^{L}}-\frac{I^{L}{\bar{I}}_{3}^{A}}{{\bar{I}}_{3}^{L}I^{A}}\right)+\frac{\delta \left(\theta +\varepsilon \right)}{\delta \theta +\varepsilon }\psi_{1}{\bar{H}}_{3}{\bar{P}}_{3}\left(3-\frac{{\bar{H}}_{3}}{H}-\frac{HP{\bar{I}}_{3}^{A}}{{\bar{H}}_{3}{\bar{P}}_{3}I^{A}}-\frac{I^{A}{\bar{P}}_{3}}{{\bar{I}}_{3}^{A}P}\right)\\ \; & +\psi_{3}\left({\bar{H}}_{3}-{\bar{H}}_{4}\right)Y^{A}. \end{array}$$

If ${\bar{ℜ}}_{4}\leq 1$, then ${\bar{\Delta }}_{4}$ does not exist because ${\bar{Y}}_{4}^{L}\leq 0$ and ${\bar{Y}}_{4}^{A}\leq 0$. In this case,

$$\begin{array}{cc} \frac{dY^{L}}{dt} & =\tau \psi_{3}HY^{A}-\left(\rho +\varpi \right)Y^{L}\leq 0,\\ \frac{dY^{A}}{dt} & =\rho Y^{L}-\varphi Y^{A}\leq 0. \end{array}$$

It follows that

$$\frac{dY^{L}}{dt}+\frac{\rho +\varpi }{\rho }\frac{dY^{A}}{dt}=\tau \psi_{3}HY^{A}-\frac{\varphi \left(\rho +\varpi \right)}{\rho }Y^{A}=\tau \psi_{3}\left(H-\frac{\varphi \left(\rho +\varpi \right)}{\rho \tau \psi_{3}}\right)Y^{A}\leq 0\;\mathrm{for}\;\mathrm{all}\;Y^{A}>0.$$

This happens only when ${\bar{H}}_{3}\leq \frac{\varphi \left(\rho +\varpi \right)}{\rho \tau \psi_{3}}={\bar{H}}_{4}$. Clearly, $\frac{d{\bar{\Gamma }}_{3}}{dt}\leq 0$ in Θ with $\frac{d{\bar{\Gamma }}_{3}}{dt}=0$ when $H={\bar{H}}_{3}$, $I^{L}={\bar{I}}_{3}^{L},$ $I^{A}={\bar{I}}_{3}^{A},$ $P={\bar{P}}_{3}$ and $Y^{A}=0.$ The solutions of system (4) tend to the invariant set ${\bar{\Pi }}_{3}^{{}^{\prime }}$ which consists of elements satisfying ${\bar{\Pi }}_{3}^{{}^{\prime }}$, we have $I^{A}\left(t\right)={\bar{I}}_{3}^{A},$ $P\left(t\right)={\bar{P}}_{3},$ $Y^{A}\left(t\right)=0$ and then $\frac{dY^{A}\left(t\right)}{dt}=0,$ $\frac{dP(t)}{dt}=0$. The fifth and sixth equations of system (4) give

$$\begin{array}{cc} 0 & =\frac{dY^{A}\left(t\right)}{dt}=\rho Y^{L}\left(t\right)⟹Y^{L}\left(t\right)=0\\ 0 & =\frac{dP(t)}{dt}=\kappa {\bar{I}}_{3}^{A}-\beta {\bar{P}}_{3}-\pi B\left(t\right){\bar{P}}_{3}⟹B\left(t\right)={\bar{B}}_{3}. \end{array}$$

Therefore, ${\bar{\Pi }}_{3}^{{}^{\prime }}=\left\{\bar{\Delta }{}_{3}\right\}$ and, from the Lyapunov–LaSalle asymptotic stability theorem, we observe that ${\bar{\Delta }}_{3}$ is GAS. □

**Theorem** **5.** If $\frac{{\bar{ℜ}}_{1}}{{\bar{ℜ}}_{2}}>1$, ${\bar{ℜ}}_{4}^{*}>1$ and ${\bar{ℜ}}_{4}>1$, then ${\bar{\Delta }}_{4}$ is GAS.

**Proof.**  Define ${\bar{\Gamma }}_{4}\left(H,I^{L},I^{A},Y^{L},Y^{A},P,B\right)$ as:

$$\begin{array}{cc} {\bar{\Gamma }}_{4} & ={\bar{H}}_{4}\Phi \left(\frac{H}{{\bar{H}}_{4}}\right)+\frac{\varepsilon }{\delta \theta +\varepsilon }{\bar{I}}_{4}^{L}\Phi \left(\frac{I^{L}}{{\bar{I}}_{4}^{L}}\right)+\frac{\theta +\varepsilon }{\delta \theta +\varepsilon }{\bar{I}}_{4}^{A}\Phi \left(\frac{I^{A}}{{\bar{I}}_{4}^{A}}\right)+\frac{1}{\tau }{\bar{Y}}_{4}^{L}\Phi \left(\frac{Y^{L}}{{\bar{Y}}_{4}^{L}}\right)\\ \; & +\frac{\rho +\varpi }{\tau \rho }{\bar{Y}}_{4}^{A}\Phi \left(\frac{Y^{A}}{{\bar{Y}}_{4}^{A}}\right)+\frac{\psi_{1}{\bar{H}}_{4}{\bar{P}}_{4}}{\kappa {\bar{I}}_{4}^{A}}{\bar{P}}_{4}\Phi \left(\frac{P}{{\bar{P}}_{4}}\right)+\frac{\pi \psi_{1}{\bar{H}}_{4}{\bar{P}}_{4}}{\eta \kappa {\bar{I}}_{4}^{A}}{\bar{B}}_{4}\Phi \left(\frac{B}{{\bar{B}}_{4}}\right). \end{array}$$

Calculating $\frac{d{\bar{\Gamma }}_{4}}{dt}$ as:

(16) $$\begin{array}{cc} \frac{d{\bar{\Gamma }}_{4}}{dt} & =\left(1-\frac{{\bar{H}}_{4}}{H}\right)\left[\xi -\alpha H-\psi_{1}HP-\psi_{2}HI^{A}-\psi_{3}HY^{A}\right]\\ \; & +\frac{\varepsilon }{\delta \theta +\varepsilon }\left(1-\frac{{\bar{I}}_{4}^{L}}{I^{L}}\right)\left[\left(1-\delta \right)\left(\psi_{1}HP+\psi_{2}HI^{A}\right)-\left(\varepsilon +\theta \right)I^{L}\right]\\ \; & +\frac{\theta +\varepsilon }{\delta \theta +\varepsilon }\left(1-\frac{{\bar{I}}_{4}^{A}}{I^{A}}\right)\left[\delta \left(\psi_{1}HP+\psi_{2}HI^{A}\right)+\varepsilon I^{L}-\gamma I^{A}\right]+\frac{1}{\tau }\left(1-\frac{{\bar{Y}}_{4}^{L}}{Y^{L}}\right)\left[\tau \psi_{3}HY^{A}-\left(\rho +\varpi \right)Y^{L}\right]\\ \; & +\frac{\rho +\varpi }{\tau \rho }\left(1-\frac{{\bar{Y}}_{4}^{A}}{Y^{A}}\right)\left[\rho Y^{L}-\varphi Y^{A}\right]+\frac{\psi_{1}{\bar{H}}_{4}{\bar{P}}_{4}}{\kappa {\bar{I}}_{4}^{A}}\left(1-\frac{{\bar{P}}_{4}}{P}\right)\left[\kappa I^{A}-\beta P-\pi BP\right]\\ \; & +\frac{\pi \psi_{1}{\bar{H}}_{4}{\bar{P}}_{4}}{\eta \kappa {\bar{I}}_{4}^{A}}\left(1-\frac{{\bar{B}}_{4}}{B}\right)\left[\eta BP-\lambda B\right]. \end{array}$$

Equation (16) can be simplified as:

$$\begin{array}{cc} \frac{d{\bar{\Gamma }}_{4}}{dt} & =\left(1-\frac{{\bar{H}}_{4}}{H}\right)\left(\xi -\alpha H\right)+\psi_{1}{\bar{H}}_{4}P+\psi_{2}{\bar{H}}_{4}I^{A}+\psi_{3}{\bar{H}}_{4}Y^{A}-\frac{\varepsilon \left(1-\delta \right)}{\delta \theta +\varepsilon }\psi_{1}HP\frac{{\bar{I}}_{4}^{L}}{I^{L}}-\frac{\varepsilon \left(1-\delta \right)}{\delta \theta +\varepsilon }\psi_{2}HI^{A}\frac{{\bar{I}}_{4}^{L}}{I^{L}}\\ \; & +\frac{\varepsilon \left(\theta +\varepsilon \right)}{\delta \theta +\varepsilon }{\bar{I}}_{4}^{L}-\frac{\gamma \left(\theta +\varepsilon \right)}{\delta \theta +\varepsilon }I^{A}-\frac{\delta \left(\theta +\varepsilon \right)}{\delta \theta +\varepsilon }\psi_{1}HP\frac{{\bar{I}}_{4}^{A}}{I^{A}}-\frac{\delta \left(\theta +\varepsilon \right)}{\delta \theta +\varepsilon }\psi_{2}H{\bar{I}}_{4}^{A}-\frac{\varepsilon \left(\theta +\varepsilon \right)}{\delta \theta +\varepsilon }I^{L}\frac{{\bar{I}}_{4}^{A}}{I^{A}}\\ \; & +\frac{\gamma \left(\theta +\varepsilon \right)}{\delta \theta +\varepsilon }{\bar{I}}_{4}^{A}-\psi_{3}HY^{A}\frac{{\bar{Y}}_{4}^{L}}{Y^{L}}+\frac{\rho +\varpi }{\tau }{\bar{Y}}_{4}^{L}-\frac{\varphi \left(\rho +\varpi \right)}{\tau \rho }Y^{A}-\frac{\rho +\varpi }{\tau }Y^{L}\frac{{\bar{Y}}_{4}^{A}}{Y^{A}}+\frac{\varphi \left(\rho +\varpi \right)}{\tau \rho }{\bar{Y}}_{4}^{A}\\ \; & +\psi_{1}{\bar{H}}_{4}{\bar{P}}_{4}\frac{I^{A}}{{\bar{I}}_{4}^{A}}-\psi_{1}{\bar{H}}_{4}{\bar{P}}_{4}\frac{\beta P}{\kappa {\bar{I}}_{4}^{A}}-\psi_{1}{\bar{H}}_{4}{\bar{P}}_{4}\frac{I^{A}{\bar{P}}_{4}}{{\bar{I}}_{4}^{A}P}+\psi_{1}{\bar{H}}_{4}{\bar{P}}_{4}\frac{\beta {\bar{P}}_{4}}{\kappa {\bar{I}}_{4}^{A}}+\psi_{1}{\bar{H}}_{4}{\bar{P}}_{4}\frac{\pi B{\bar{P}}_{4}}{\kappa {\bar{I}}_{4}^{A}}-\psi_{1}{\bar{H}}_{4}{\bar{P}}_{4}\frac{\pi \lambda B}{\eta \kappa {\bar{I}}_{4}^{A}}\\ \; & -\psi_{1}{\bar{H}}_{4}{\bar{P}}_{4}\frac{\pi {\bar{B}}_{4}P}{\kappa {\bar{I}}_{4}^{A}}+\psi_{1}{\bar{H}}_{4}{\bar{P}}_{4}\frac{\pi \lambda {\bar{B}}_{4}}{\eta \kappa {\bar{I}}_{4}^{A}}. \end{array}$$

The steady state conditions for ${\bar{\Delta }}_{4}$ yield

$$\begin{array}{cc} \; & \left.\xi =\alpha {\bar{H}}_{4}+\psi_{1}{\bar{H}}_{4}{\bar{P}}_{4}+\psi_{2}{\bar{H}}_{4}{\bar{I}}_{4}^{A}+\psi_{3}{\bar{H}}_{4}{\bar{Y}}_{4}^{A},\right.\\ \; & \left.\frac{\varepsilon \left(1-\delta \right)}{\delta \theta +\varepsilon }\left(\psi_{1}{\bar{H}}_{4}{\bar{P}}_{4}+\psi_{2}{\bar{H}}_{4}{\bar{I}}_{4}^{A}\right)=\frac{\varepsilon \left(\theta +\varepsilon \right)}{\delta \theta +\varepsilon }{\bar{I}}_{4}^{L},\right.\\ \; & \left.\psi_{1}{\bar{H}}_{4}{\bar{P}}_{4}+\psi_{2}{\bar{H}}_{4}{\bar{I}}_{4}^{A}=\frac{\gamma \left(\theta +\varepsilon \right)}{\delta \theta +\varepsilon }{\bar{I}}_{4}^{A},\;\;\;\;{\bar{P}}_{4}=\frac{\lambda }{\eta },\right.\\ \; & \left.\psi_{3}{\bar{H}}_{4}{\bar{Y}}_{4}^{A}=\frac{\rho +\varpi }{\tau }{\bar{Y}}_{4}^{L}=\frac{\varphi \left(\rho +\varpi \right)}{\tau \rho }{\bar{Y}}_{4}^{A},\;\;\;\kappa {\bar{I}}_{4}^{A}=\beta {\bar{P}}_{4}+\pi {\bar{B}}_{4}{\bar{P}}_{4}.\right. \end{array}$$

Then, we obtain

$$\begin{array}{cc} \frac{d{\bar{\Gamma }}_{4}}{dt} & =\left(1-\frac{{\bar{H}}_{4}}{H}\right)\left(\alpha {\bar{H}}_{4}-\alpha H\right)+\left(\psi_{1}{\bar{H}}_{4}{\bar{P}}_{4}+\psi_{2}{\bar{H}}_{4}{\bar{I}}_{4}^{A}+\psi_{3}{\bar{H}}_{4}{\bar{Y}}_{4}^{A}\right)\left(1-\frac{{\bar{H}}_{4}}{H}\right)-\frac{\varepsilon \left(1-\delta \right)}{\delta \theta +\varepsilon }\psi_{1}{\bar{H}}_{4}{\bar{P}}_{4}\frac{HP{\bar{I}}_{4}^{L}}{{\bar{H}}_{4}{\bar{P}}_{4}I^{L}}\\ \; & -\frac{\varepsilon \left(1-\delta \right)}{\delta \theta +\varepsilon }\psi_{2}{\bar{H}}_{4}{\bar{I}}_{4}^{A}\frac{HI^{A}{\bar{I}}_{4}^{L}}{{\bar{H}}_{4}{\bar{I}}_{4}^{A}I^{L}}+\frac{\varepsilon \left(1-\delta \right)}{\delta \theta +\varepsilon }\left(\psi_{1}{\bar{H}}_{4}{\bar{P}}_{4}+\psi_{2}{\bar{H}}_{4}{\bar{I}}_{4}^{A}\right)-\frac{\delta \left(\theta +\varepsilon \right)}{\delta \theta +\varepsilon }\psi_{1}{\bar{H}}_{4}{\bar{P}}_{4}\frac{HP{\bar{I}}_{4}^{A}}{{\bar{H}}_{4}{\bar{P}}_{4}I^{A}}\\ \; & -\frac{\delta \left(\theta +\varepsilon \right)}{\delta \theta +\varepsilon }\psi_{2}{\bar{H}}_{4}{\bar{I}}_{4}^{A}\frac{H}{{\bar{H}}_{4}}-\frac{\varepsilon \left(1-\delta \right)}{\delta \theta +\varepsilon }\left(\psi_{1}{\bar{H}}_{4}{\bar{P}}_{4}+\psi_{2}{\bar{H}}_{4}{\bar{I}}_{4}^{A}\right)\frac{I^{L}{\bar{I}}_{4}^{A}}{{\bar{I}}_{4}^{L}I^{A}}+\psi_{1}{\bar{H}}_{4}{\bar{P}}_{4}+\psi_{2}{\bar{H}}_{4}{\bar{I}}_{4}^{A}\\ \; & -\psi_{3}{\bar{H}}_{4}{\bar{Y}}_{4}^{A}\frac{HY^{A}{\bar{Y}}_{4}^{L}}{{\bar{H}}_{4}{\bar{Y}}_{4}^{A}Y^{L}}+\psi_{3}{\bar{H}}_{4}{\bar{Y}}_{4}^{A}-\psi_{3}{\bar{H}}_{4}{\bar{Y}}_{4}^{A}\frac{Y^{L}{\bar{Y}}_{4}^{A}}{{\bar{Y}}_{4}^{L}Y^{A}}+\psi_{3}{\bar{H}}_{4}{\bar{Y}}_{4}^{A}-\psi_{1}{\bar{H}}_{4}{\bar{P}}_{4}\frac{I^{A}{\bar{P}}_{4}}{{\bar{I}}_{4}^{A}P}+\psi_{1}{\bar{H}}_{4}{\bar{P}}_{4}\\ \; & =-\left[\alpha +\frac{\delta \psi_{2}{\bar{I}}_{4}^{A}\left(\theta +\varepsilon \right)}{\delta \theta +\varepsilon }\right]\frac{{\left(H-{\bar{H}}_{4}\right)}^{2}}{H}+\frac{\varepsilon \left(1-\delta \right)}{\delta \theta +\varepsilon }\psi_{1}{\bar{H}}_{4}{\bar{P}}_{4}\left(4-\frac{{\bar{H}}_{4}}{H}-\frac{HP{\bar{I}}_{4}^{L}}{{\bar{H}}_{4}{\bar{P}}_{4}I^{L}}-\frac{I^{L}{\bar{I}}_{4}^{A}}{{\bar{I}}_{4}^{L}I^{A}}-\frac{I^{A}{\bar{P}}_{4}}{{\bar{I}}_{4}^{A}P}\right)\\ \; & +\frac{\varepsilon \left(1-\delta \right)}{\delta \theta +\varepsilon }\psi_{2}{\bar{H}}_{4}{\bar{I}}_{4}^{A}\left(3-\frac{{\bar{H}}_{4}}{H}-\frac{HI^{A}{\bar{I}}_{4}^{L}}{{\bar{H}}_{4}{\bar{I}}_{4}^{A}I^{L}}-\frac{I^{L}{\bar{I}}_{4}^{A}}{{\bar{I}}_{4}^{L}I^{A}}\right)+\frac{\delta \left(\theta +\varepsilon \right)}{\delta \theta +\varepsilon }\psi_{1}{\bar{H}}_{4}{\bar{P}}_{4}\left(3-\frac{{\bar{H}}_{4}}{H}-\frac{HP{\bar{I}}_{4}^{A}}{{\bar{H}}_{4}{\bar{P}}_{4}I^{A}}-\frac{I^{A}{\bar{P}}_{4}}{{\bar{I}}_{4}^{A}P}\right)\\ \; & +\psi_{3}{\bar{H}}_{4}{\bar{Y}}_{4}^{A}\left(3-\frac{{\bar{H}}_{4}}{H}-\frac{HY^{A}{\bar{Y}}_{4}^{L}}{{\bar{H}}_{4}{\bar{Y}}_{4}^{A}Y^{L}}-\frac{Y^{L}{\bar{Y}}_{4}^{A}}{{\bar{Y}}_{4}^{L}Y^{A}}\right). \end{array}$$

If ${\bar{ℜ}}_{1}/{\bar{ℜ}}_{2}>1$, ${\bar{ℜ}}_{4}^{*}>1$ and ${\bar{ℜ}}_{4}>1$, then $\frac{d{\bar{\Gamma }}_{4}}{dt}\leq 0$ in $\overset{˚}{\Theta }$, where $\overset{˚}{\Theta }$ is the interior of Θ. Similarly to the previous Theorems, one can show that $\frac{d{\bar{\Gamma }}_{4}}{dt}=0$ when $H={\bar{H}}_{4}$, $I^{L}={\bar{I}}_{4}^{L},$ $I^{A}={\bar{I}}_{4}^{A},$ $Y^{L}={\bar{Y}}_{4}^{L},$ $Y^{A}={\bar{Y}}_{4}^{A}$ and $P={\bar{P}}_{4}.$ The solutions of system (4) tend to the invariant set ${\bar{\Pi }}_{4}^{{}^{\prime }}$ with $I^{A}\left(t\right)={\bar{I}}_{4}^{A}$, $P\left(t\right)={\bar{P}}_{4}$ and then $\frac{dP(t)}{dt}=0$. The sixth equations of system (4) imply that

$$0=\frac{dP(t)}{dt}=\kappa {\bar{I}}_{4}^{A}-\beta {\bar{P}}_{4}-\pi B\left(t\right){\bar{P}}_{4},$$

which gives $B\left(t\right)={\bar{B}}_{4}$ and hence ${\bar{\Pi }}_{4}^{{}^{\prime }}=\left\{{\bar{\Delta }}_{4}\right\}$. Applying the Lyapunov–LaSalle asymptotic stability theorem, we can observe that ${\bar{\Delta }}_{4}$ is GAS. □

In Table 2, we present the global stability conditions for all steady states of model (4).

## 3. Numerical Simulations

In this section, we conduct numerical simulations to illustrate the results given in Theorems 1–5. Furthermore, we investigate the impact of HIV-1-specific antibodies on HIV-1/HTLV-I co-infection dynamics. We solve system (4) numerically using the values of the parameters given in Table 3. Furthermore, the variation of some parameter values that have a significant effect on the threshold parameters and thus the stability behavior will be used to demonstrate the analytic results acquired above. The values of some parameters are taken form the literature. The other parameters have been chosen merely to perform the numerical simulations. This is due to the lack of real data from HIV-1/HTLV-I co-infected individuals; however, if one has real data then the parameters of the model can be estimated and the validity of the model can be established.

### 3.1. Stability of the Steady States

The analytical results from Section 2 are illustrated here. The following initial conditions are used to ensure that each initial point in the feasible set leads to just one steady state in our system’s solution.

**IS-1:**$\left(H,I^{L},I^{A},Y^{L},Y^{A},P,B\right)\left(0\right)=\left(600,0.5,1.5,1,2,5,1\right)$,

**IS-2:** $\left(H,I^{L},I^{A},Y^{L},Y^{A},P,B\right)\left(0\right)=\left(400,1,1,1.5,4,2,2\right),$

**IS-3:**$\left(H,I^{L},I^{A},Y^{L},Y^{A},P,B\right)\left(0\right)=\left(200,1.5,0.5,2,6,1.5,3\right)$.

**Figure 1:**
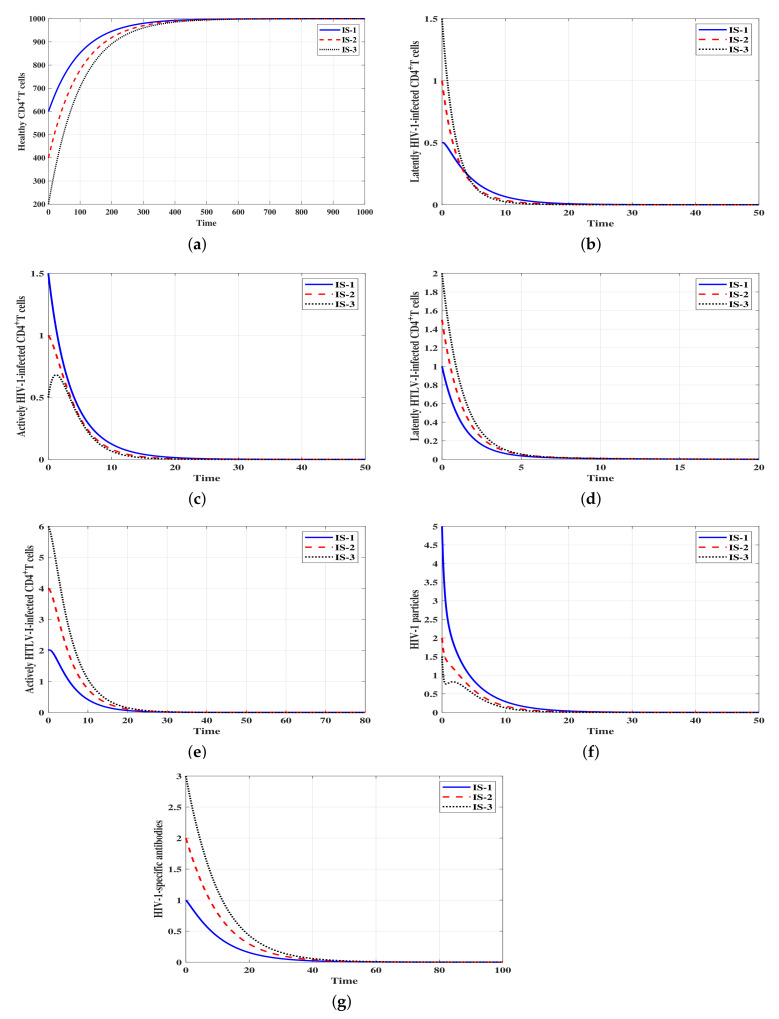
Solutions of system (4) with different initial conditions and when ${\bar{ℜ}}_{1}\leq 1$ and ${\bar{ℜ}}_{2}\leq 1$. The steady state ${\bar{\Delta }}_{0}=\left(1000,0,0,0,0,0,0\right)$ is GAS. (**a**) Healthy CD4${}^{+}$ T cells, (**b**) Latent HIV-1-infected CD4${}^{+}$ T cells, (**c**) Active HIV-1-infected CD4${}^{+}$ T cells, (**d**) Latent HTLV-I-infected CD4${}^{+}$ T cells, (**e**) Active HTLV-I-infected CD4${}^{+}$ T cells, (**f**) HIV-1 particles, (**g**) HIV-1-specific antibodies.

**Figure 2:**
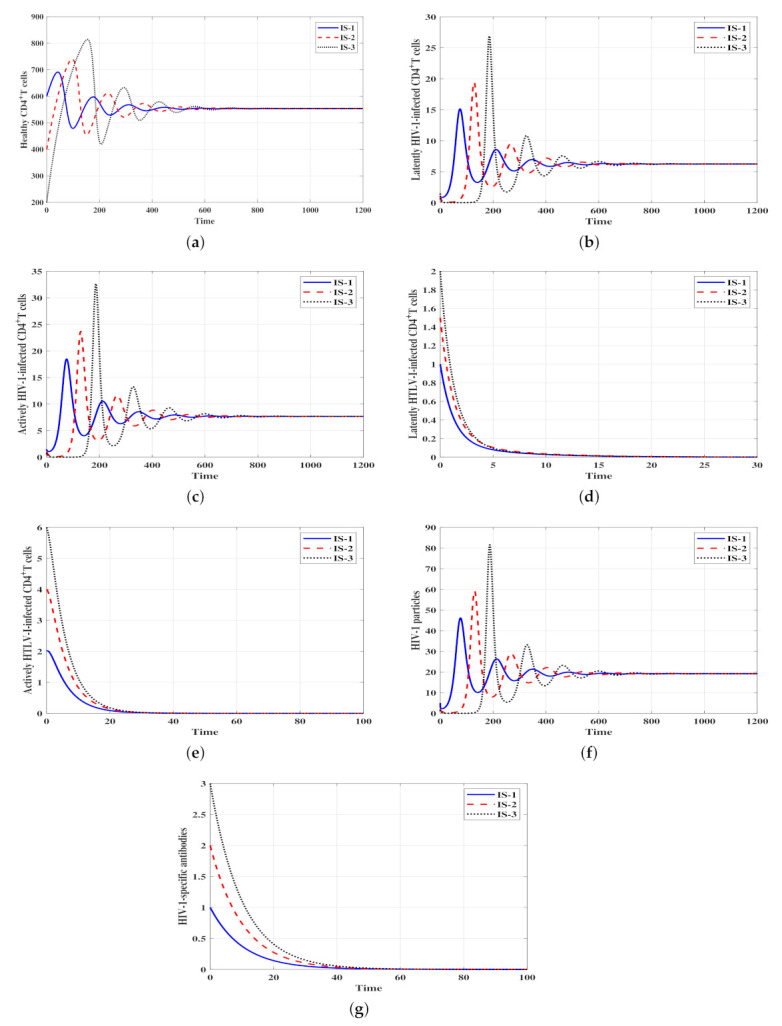
Solutions of system (4) with different initial conditions and when ${\bar{ℜ}}_{1}>1$, $\frac{{\bar{ℜ}}_{2}}{{\bar{ℜ}}_{1}}\leq 1$ and ${\bar{ℜ}}_{3}\leq 1$. The steady state ${\bar{\Delta }}_{1}=\left(553.71,6.25,7.68,0,0,19.2,0\right)$ is GAS. (**a**) Healthy CD4${}^{+}$ T cells, (**b**) Latent HIV-1-infected CD4${}^{+}$ T cells, (**c**) Active HIV-1-infected CD4${}^{+}$ T cells, (**d**) Latent HTLV-I-infected CD4${}^{+}$ T cells, (**e**) Active HTLV-I-infected CD4${}^{+}$ T cells, (**f**) HIV-1 particles, (**g**) HIV-1-specific antibodies.

**Figure 3:**
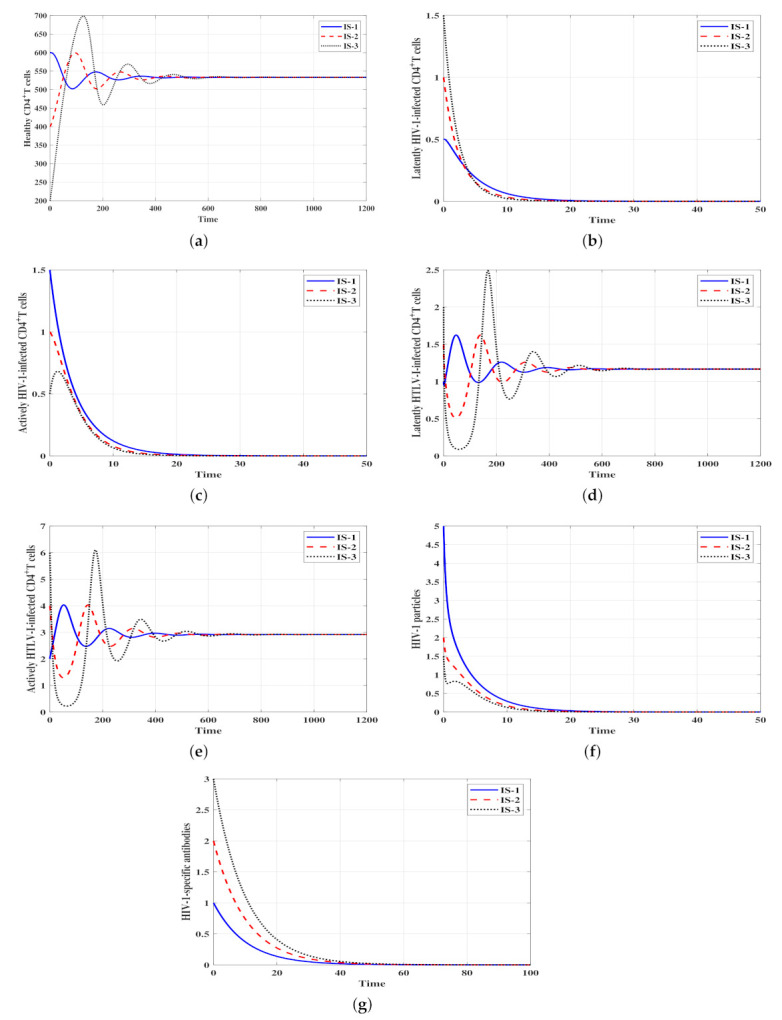
Solutions of system (4) with different initials and when ${\bar{ℜ}}_{2}>1$, $\frac{{\bar{ℜ}}_{1}}{{\bar{ℜ}}_{2}}\leq 1$. The steady state ${\bar{\Delta }}_{2}=\left(533.33,0,0,1.17,2.92,0,0\right)$ is GAS. (**a**) Healthy CD4${}^{+}$ T cells, (**b**) Latent HIV-1-infected CD4${}^{+}$ T cells, (**c**) Active HIV-1-infected CD4${}^{+}$ T cells, (**d**) Latent HTLV-I-infected CD4${}^{+}$ T cells, (**e**) Active HTLV-I-infected CD4${}^{+}$ T cells, (**f**) HIV-1 particles, (**g**) HIV-1-specific antibodies.

**Figure 4:**
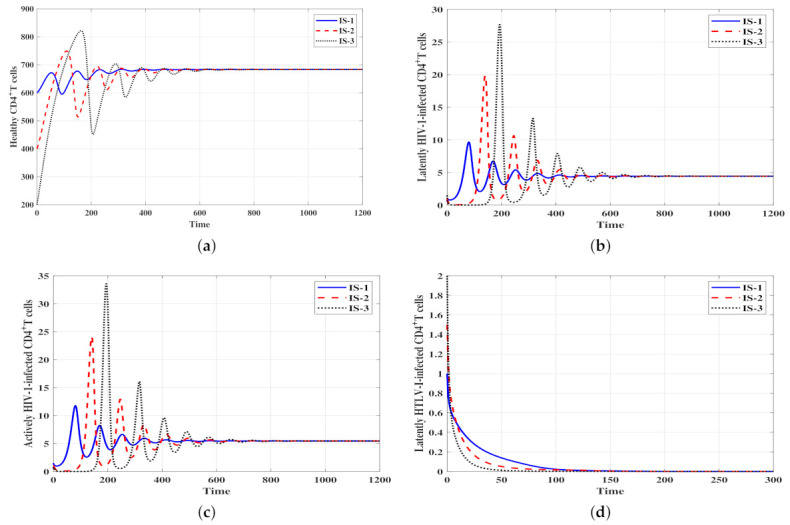

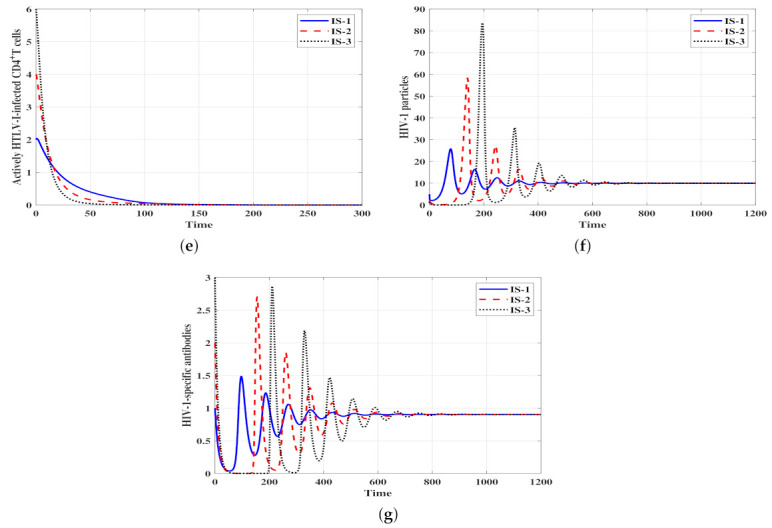
Solutions of system (4) with different initial conditions and when ${\bar{ℜ}}_{3}>1$ and ${\bar{ℜ}}_{4}\leq 1$. The steady state ${\bar{\Delta }}_{3}=\left(683.34,4.43,5.45,0,0,10,0.9\right)$ is GAS. (**a**) Healthy CD4${}^{+}$ T cells, (**b**) Latent HIV-1-infected CD4${}^{+}$ T cells, (**c**) Active HIV-1-infected CD4${}^{+}$ T cells, (**d**) Latent HTLV-I-infected CD4${}^{+}$ T cells, (**e**) Active HTLV-I-infected CD4${}^{+}$ T cells, (**f**) HIV-1 particles, (**g**) HIV-1-specific antibodies.

**Figure 5:**
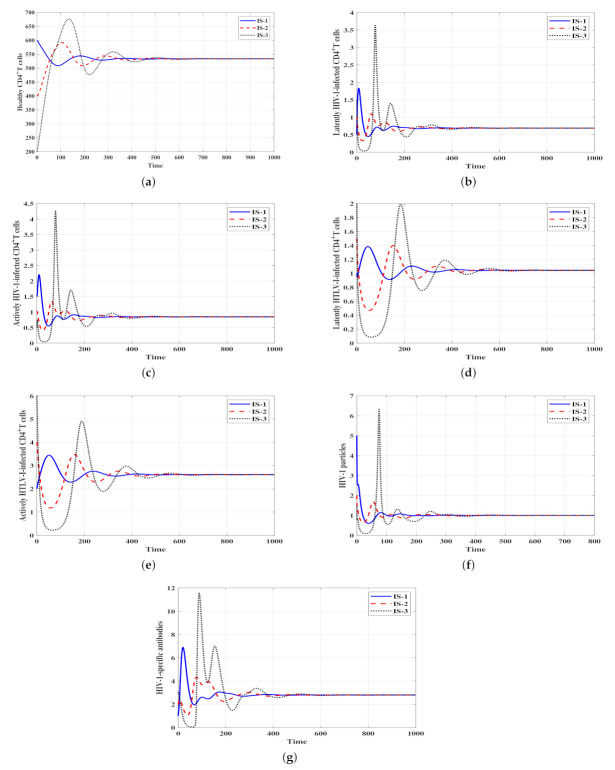
Solutions of system (4) with different initial conditions and when ${\bar{ℜ}}_{4}^{*}>1,$ ${\bar{ℜ}}_{4}>1$ and $\frac{{\bar{ℜ}}_{1}}{{\bar{ℜ}}_{2}}>1$. The steady state ${\bar{\Delta }}_{4}=\left(533.33,0.69,0.85,1.04,2.61,1,2.8\right)$ is GAS. (**a**) Healthy CD4${}^{+}$ T cells, (**b**) Latent HIV-1-infected CD4${}^{+}$ T cells, (**c**) Active HIV-1-infected CD4${}^{+}$ T cells, (**d**) Latent HTLV-I-infected CD4${}^{+}$ T cells, (**e**) Active HTLV-I-infected CD4${}^{+}$ T cells, (**f**) HIV-1 particles, (**g**) HIV-1-specific antibodies.

Selecting different values of $\psi_{1}$,$\psi_{2},$ $\psi_{3}$ and η leads to the following cases:

- Stability of ${\bar{\Delta }}_{0}$: $\psi_{1}=\psi_{2}=\psi_{3}=0.0001$ and η=0.01. With these values, we obtain ${\bar{ℜ}}_{1}=0.6<1$ and ${\bar{ℜ}}_{2}=0.06<1$. Figure 1 shows that the solutions of the model with the three initial conditions IS-1, IS-2 and IS-3 converge to the infection-free steady state ${\bar{\Delta }}_{0}=\left(1000,0,0,0,0,0,0\right)$. The numerical results shown in Figure 1 illustrate the results of Theorem 1. This result suggests that when ${\bar{ℜ}}_{1}\leq 1$ and ${\bar{ℜ}}_{2}\leq 1$ both HIV-1 and HTLV-I are predicted to die out, regardless of the initial conditions. From a control viewpoint, making ${\bar{ℜ}}_{1}\leq 1$ and ${\bar{ℜ}}_{2}\leq 1$ will be an ideal approach, but HTLV-I and HIV-1 infections are lifelong, and the viruses are rarely cleared.
- Stability of ${\bar{\Delta }}_{1}$: $\psi_{1}=\psi_{2}=\psi_{3}=0.0003$ and η=0.001. We obtain ${\bar{ℜ}}_{1}=1.8$, ${\bar{ℜ}}_{2}=0.18,$ ${\bar{ℜ}}_{3}=0.3<1$ and hence $\frac{{\bar{ℜ}}_{2}}{{\bar{ℜ}}_{1}}=0.1<1$. Therefore, the stability conditions given in Theorem 2 are satisfied and the infected HIV-1 mono-infection steady state with inefficacious humoral immunity ${\bar{\Delta }}_{1}$ is GAS. In Figure 2, we can observe that the solutions of the model with the three initial conditions IS-1, IS-2 and IS-3 tend to ${\bar{\Delta }}_{1}=\left(553.71,6.25,7.68,0,0,19.2,0\right)$. This result suggests that HTLV-I will die out, whereas HIV-1 will be chronic with ineffective humoral immunity.
- Stability of ${\bar{\Delta }}_{2}$: $\psi_{1}=\psi_{2}=0.0001,$ $\psi_{3}=0.003$ and η=0.001. These values of parameters yield ${\bar{ℜ}}_{1}=0.6,$ ${\bar{ℜ}}_{2}=1.9$, ${\bar{ℜ}}_{4}=0.9$ and then $\frac{{\bar{ℜ}}_{1}}{{\bar{ℜ}}_{2}}=0.32<1$. Hence, Theorem 3 is applicable and the infected HTLV-I mono-infection steady state ${\bar{\Delta }}_{2}$ is GAS. Figure 3 shows that the solutions of model (4) with initial conditions IS-1, IS-2 and IS-3 lead to the steady state ${\bar{\Delta }}_{2}=\left(533.33,0,0,1.17,2.92,0,0\right)$. This observation is consistent with the outcomes of Theorem 3. This result suggests that HIV-1 will die out, whereas HTLV-I will be chronic.
- Stability of ${\bar{\Delta }}_{3}$: $\psi_{1}=\psi_{2}=0.0003,$ $\psi_{3}=0.002$ and η=0.01. With these values we obtain ${\bar{ℜ}}_{3}=1.4>1$ and ${\bar{ℜ}}_{4}=0.8<1$. Hence, the stability conditions of Theorem 4 are valid and the infected HIV-1 mono-infection steady state with efficacious humoral immunity ${\bar{\Delta }}_{3}$ is GAS. In Figure 4, we can observe that the solutions of the system with the three initial conditions IS-1, IS-2 and IS-3 tend to ${\bar{\Delta }}_{3}=\left(683.34,4.43,5.45,0,0,10,0.9\right)$. This result indicates that HTLV-I is predicted to die out, whereas HIV-1 will be chronic with active humoral immunity.
- Stability of ${\bar{\Delta }}_{4}$: $\psi_{1}=\psi_{2}=0.0005,$ $\psi_{3}=0.003$ and η=0.1. Then, we calculate ${\bar{ℜ}}_{4}^{*}=2.18>1,$ ${\bar{ℜ}}_{4}=1.72>1$ and $\frac{{\bar{ℜ}}_{1}}{{\bar{ℜ}}_{2}}=1.6>1$. Therefore, the result of Theorem 5 is valid and the infected HIV-1/HTLV-I co-infection steady state with efficacious humoral immunity ${\bar{\Delta }}_{4}$ is GAS. Figure 5 illustrates that starting from the three initial conditions, IS-1, IS-2 and IS-3, the solutions of the system converge to ${\bar{\Delta }}_{4}=\left(533.33,0.69,0.85,1.04,2.61,1,2.8\right)$. This result suggests that both HTLV-I and HIV-1 will be chronic with active humoral immune response.

### 3.2. Effect of Humoral Immunity on the HIV-1/HTLV-I Co-Infection Dynamics

In this subsection, we study the impact of HIV-1-specific antibodies on the HIV-1/HTLV-I co-infection dynamics. We note that the stability of the infection-free steady state ${\bar{\Delta }}_{0}$ depends on the parameters ${\bar{ℜ}}_{1}$ and ${\bar{ℜ}}_{2}$. These parameters do not depend on the proliferation of the HIV-1-specific antibodies η. Therefore, HIV-1-specific antibodies do not play the role of clearing the HIV-1 infection, but they have an important role in controlling and suppressing HTLV-I infection. To observe the effect of HIV-1-specific antibodies on the solutions of the model, we fixed the parameters $\psi_{1}=\psi_{2}=0.0005,$ $\psi_{3}=0.003$ and varied the parameterη. We chose the following initial conditions:

**IS-4:**$\left(H,I^{L},I^{A},Y^{L},Y^{A},P,B\right)\left(0\right)=\left(500,3,1.5,0.8,3,2,3\right)$.

**Figure 6:**
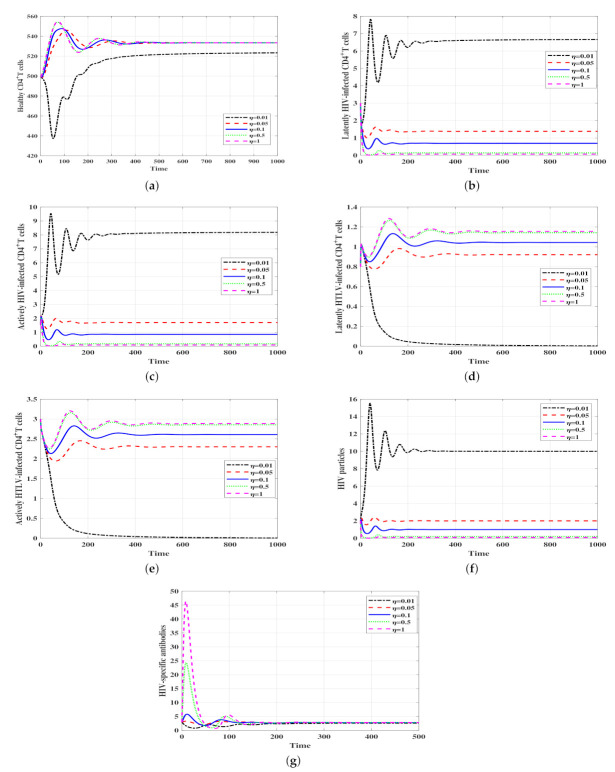
Effect of HIV-1-specific antibodies on HIV-1/HTLV-I co-infection dynamics. (**a**) Healthy CD4${}^{+}$ T cells, (**b**) Latent HIV-1-infected CD4${}^{+}$ T cells, (**c**) Active HIV-1-infected CD4${}^{+}$ T cells, (**d**) Latent HTLV-I-infected CD4${}^{+}$ T cells, (**e**) Active HTLV-I-infected CD4${}^{+}$ T cells, (**f**) HIV-1 particles, (**g**) HIV-1-specific antibodies.

We can see from Figure 6 that when η is increased, the concentrations of HIV-1 particles and latent/active HIV-1-infected CD$4^{+}$ T cells are decreased, whereas the concentrations of latent/active HTLV-I-infected CD$4^{+}$ T cells are increased. Therefore, HIV-1-specific antibodies can control HIV-1 infection, but they may enhance the progression of HTLV-I.

## 4. Conclusions and Discussion

In this article, we have studied a within-host HIV-1/HTLV-I co-infection model with humoral immunity with both V-T-C and C-T-C modes of transmission. We have presented some preliminary results regarding the positivity and boundedness of the models’ solutions. By constructing suitable Lyapunov functions and using LaSalle’s invariance principle, we have identified four threshold parameters for the global stability of steady states. More precisely, it has been shown that, if ${\bar{ℜ}}_{1}\leq 1$ and ${\bar{ℜ}}_{2}\leq 1$, then the infection-free steady state ${\bar{\Delta }}_{0}$ is GAS; if ${\bar{ℜ}}_{1}>1,$ $\frac{{\bar{ℜ}}_{2}}{{\bar{ℜ}}_{1}}\leq 1$ and ${\bar{ℜ}}_{3}\leq 1$, then the infected HIV-1 mono-infection steady state with inefficacious humoral immunity ${\bar{\Delta }}_{1}$ is GAS; if ${\bar{ℜ}}_{2}>1$ and $\frac{{\bar{ℜ}}_{1}}{{\bar{ℜ}}_{2}}\leq 1$, then the infected HTLV-I mono-infection steady state ${\bar{\Delta }}_{2}$ is GAS; If ${\bar{ℜ}}_{3}>1$ and ${\bar{ℜ}}_{4}\leq 1$, then the infected HIV-1 mono-infection steady state with efficacious humoral immunity ${\bar{\Delta }}_{3}$ is GAS; and if $\frac{{\bar{ℜ}}_{1}}{{\bar{ℜ}}_{2}}>1$, ${\bar{ℜ}}_{4}^{*}>1$ and ${\bar{ℜ}}_{4}>1$, then the infected HIV-1/HTLV-I co-infection steady state with efficacious humoral immunity ${\bar{\Delta }}_{4}$ is GAS. Numerical simulations have been provided to show the strength and credibility of our theoretical results.

Let us consider the case when C-T-C transmission is omitted in the HIV-1 replication model. Then, the HIV-1/HTLV-I co-infection model with humoral immunity is given as:

(17) $$\left\{\begin{array}{c} \frac{dH}{dt}=\xi -\alpha H-\psi_{1}HP-\psi_{3}HY^{A},\\ \frac{dI^{L}}{dt}=\left(1-\delta \right)\psi_{1}HP-\left(\varepsilon +\theta \right)I^{L},\\ \frac{dI^{A}}{dt}=\delta \psi_{1}HP+\varepsilon I^{L}-\gamma I^{A},\\ \frac{dY^{L}}{dt}=\tau \psi_{3}HY^{A}-\left(\rho +\varpi \right)Y^{L},\\ \frac{dY^{A}}{dt}=\rho Y^{L}-\varphi Y^{A},\\ \frac{dP}{dt}=\kappa I^{A}-\beta P-\pi BP,\\ \frac{dB}{dt}=\eta BP-\lambda B. \end{array}\right.$$

Model (17) has an infection-free steady state $\Delta_{0}={\bar{\Delta }}_{0}$ and it is GAS when the two threshold parameters ${ℜ}_{1}\leq 1$ and ${ℜ}_{2}\leq 1$, where

$${ℜ}_{1}=\frac{H_{0}\kappa \psi_{1}\left(\delta \theta +\varepsilon \right)}{\gamma \beta \left(\theta +\varepsilon \right)}={\bar{ℜ}}_{11}<{\bar{ℜ}}_{1},$$

and

$${ℜ}_{2}={\bar{ℜ}}_{2}=\frac{\tau \psi_{3}\rho {\bar{H}}_{0}}{\varphi \left(\rho +\varpi \right)}.$$

Here ${ℜ}_{1}$ denotes the basic HIV-1 mono-infection reproductive ratio for system (17) that corresponds to V-T-C only. Let us consider ${ℜ}_{2}\leq 1$. We note that the incorporation of C-T-C transmission into the dynamics causes an increase in the parameter ${\bar{ℜ}}_{1}$, since ${\bar{ℜ}}_{1}={\bar{ℜ}}_{11}+{\bar{ℜ}}_{12}>{\bar{ℜ}}_{11}$. As a consequence, the omission of C-T-C transmission from the HIV-1/HTLV-I co-infection model will cause an under-evaluation of the basic HIV-1 mono-infection reproductive ratio.

## Figures and Tables

**Table 1:** A summary of all variables and parameters used in model (4).

Symbol	Biological Description
Populations	
*H*	Healthy CD$4^{+}$ T cells
$I^{L}$	Latent HIV-1-infected CD$4^{+}$ T cells
$I^{A}$	Active HIV-1-infected CD$4^{+}$ T cells
$Y^{L}$	Latent HTLV-I-infected CD4${}^{+}$ T cells
$Y^{A}$	Active HTLV-I-infected CD4${}^{+}$ T cells
*P*	HIV-1 particles
*B*	HIV-1-specific antibodies
Parameters	
ξ	Rate of supply of healthy CD$4^{+}$ T cells
α	Death rates of healthy CD$4^{+}$ T cells
$\psi_{1}$	$\begin{array}{c} \mathrm{Viral}\;\mathrm{infection}\;\mathrm{rate}\;\mathrm{from}\;\mathrm{the}\;\mathrm{contact}\;\mathrm{between}\;\mathrm{HIV}\text{-}1\;\mathrm{particles}\\ \mathrm{and}\;\mathrm{healthy}\;\mathrm{CD}4^{+}\;T\;\mathrm{cells} \end{array}$
$\psi_{2}$	$\begin{array}{c} \mathrm{Cellular}\;\mathrm{infection}\;\mathrm{rate}\;\mathrm{from}\;\mathrm{the}\;\mathrm{contact}\;\mathrm{between}\;\mathrm{active}\;\mathrm{HIV}\text{-}1\text{-}\mathrm{infected}\\ \mathrm{cells}\;\mathrm{and}\;\mathrm{healthy}\;\mathrm{CD}4^{+}\;T\;\mathrm{cells} \end{array}$
$\psi_{3}$	$\begin{array}{c} \mathrm{Cellular}\;\mathrm{infection}\;\mathrm{rate}\;\mathrm{from}\;\mathrm{the}\;\mathrm{contact}\;\mathrm{between}\;\mathrm{active}\;\mathrm{HTLV}\text{-}I\text{-}\mathrm{infected}\\ \mathrm{cells}\;\mathrm{and}\;\mathrm{healthy}\;\mathrm{CD}4^{+}\;T\;\mathrm{cells} \end{array}$
$\delta \in \left(0,1\right)$	$\begin{array}{c} \mathrm{Fraction}\;\mathrm{coefficient}\;\mathrm{refers}\;\mathrm{to}\;\mathrm{the}\;\mathrm{probability}\;\mathrm{of}\;\mathrm{new}\;\mathrm{HIV}\text{-}1\text{-}\mathrm{infected}\\ \mathrm{cells}\;\mathrm{could}\;\mathrm{be}\;\mathrm{active},\;\mathrm{and}\;\mathrm{the}\;\mathrm{remaining}\;\mathrm{part}\;1-\delta \;\mathrm{will}\;\mathrm{be}\;\mathrm{silent} \end{array}$
ε	Activation rates of latent HIV-1-infected CD$4^{+}$ T cells
θ	Death rates of latent HIV-1-infected CD$4^{+}$ T cells
γ	Death rates of active HIV-1-infected CD$4^{+}$ T cells
$\tau \in \left(0,1\right)$	Probability of new HTLV-I infections could be enter a latent period
ρ	Activation rates of latent HTLV−I−infected CD$4^{+}$ T cells
ϖ	Death rates of latent HTLV−I−infected CD$4^{+}$ T cells
φ	Death rates of active HTLV−I−infected CD$4^{+}$ T cells
κ	Rate of free HIV-1 particles production
β	Death rates of HIV-1 particles
π	Neutralization rate of HIV-1 particles by HIV-1-specific antibodies
η	Proliferation rate for HIV-1-specific antibodies
λ	Death rates of HIV-1-specific antibodies

**Table 2:** Steady states of model (4) and the conditions for their existence and global stability.

Steady State	ExistenceConditions	Global Stability Conditions
${\bar{\Delta }}_{0}=\left({\bar{H}}_{0},0,0,0,0,0,0\right)$	None	${\bar{ℜ}}_{1}\leq 1$ and ${\bar{ℜ}}_{2}\leq 1$
${\bar{\Delta }}_{1}=\left({\bar{H}}_{1},{\bar{I}}_{1}^{L},{\bar{I}}_{1}^{A},0,0,{\bar{P}}_{1},0\right)$	${\bar{ℜ}}_{1}>1$	${\bar{ℜ}}_{1}>1,$$\frac{{\bar{ℜ}}_{2}}{{\bar{ℜ}}_{1}}\leq 1$ and ${\bar{ℜ}}_{3}\leq 1$
${\bar{\Delta }}_{2}=\left({\bar{H}}_{2},0,0,{\bar{Y}}_{2}^{L},{\bar{Y}}_{2}^{A},0,0\right)$	${\bar{ℜ}}_{2}>1$	${\bar{ℜ}}_{2}>1$ and $\frac{{\bar{ℜ}}_{1}}{{\bar{ℜ}}_{2}}\leq 1$
${\bar{\Delta }}_{3}=\left({\bar{H}}_{3},{\bar{I}}_{3}^{L},{\bar{I}}_{3}^{A},0,0,{\bar{P}}_{3},{\bar{B}}_{3}\right)$	${\bar{ℜ}}_{3}>1$	${\bar{ℜ}}_{3}>1$ and ${\bar{ℜ}}_{4}\leq 1$
${\bar{\Delta }}_{4}=\left({\bar{H}}_{4},{\bar{I}}_{4}^{L},{\bar{I}}_{4}^{A},{\bar{Y}}_{4}^{L},{\bar{Y}}_{4}^{A},{\bar{P}}_{4},{\bar{B}}_{4}\right)$	${\bar{ℜ}}_{4}^{*},{\bar{ℜ}}_{4}>1$ and $\frac{{\bar{ℜ}}_{1}}{{\bar{ℜ}}_{2}}>1$	$\frac{{\bar{ℜ}}_{1}}{{\bar{ℜ}}_{2}}>1$, ${\bar{ℜ}}_{4}^{*}>1$ and ${\bar{ℜ}}_{4}>1$

**Table 3:** The values of the parameters of system (4).

Parameter	Value	Source	Parameter	Value	Source
ξ	10	[49,63,64]	τ	0.2	[37]
α	0.01	[63,65,66]	ρ	0.5	assumed
$\psi_{1}$	Varied	-	ϖ	0.3	assumed
$\psi_{2}$	Varied	-	φ	0.2	[47,50,51]
$\psi_{3}$	Varied	-	κ	5	[63]
δ	0.3	[67]	β	2	[63]
ε	0.4	assumed	π	0.8	assumed
θ	0.1	[67]	η	Varied	-
γ	0.5	[68,69,70]	λ	0.1	[71]

## Data Availability

Not applicable.
